# Evolutionary history of two cryptic species of northern African jerboas

**DOI:** 10.1186/s12862-020-1592-z

**Published:** 2020-02-13

**Authors:** Ana Filipa Moutinho, Nina Serén, Joana Paupério, Teresa Luísa Silva, Fernando Martínez-Freiría, Graciela Sotelo, Rui Faria, Tapio Mappes, Paulo Célio Alves, José Carlos Brito, Zbyszek Boratyński

**Affiliations:** 1 Department of Evolutionary Genetics, Max Planck Institute for Evolutionary Biology, Plön, Germany; 2 CIBIO-InBIO Associate Laboratory, Research Centre in Biodiversity and Genetic Resources, University of Porto, Vairão, 4485-661 Vairão, Portugal; 3 Department of Biology, Faculty of Science, University of Porto, Porto, Portugal; 4 Division of Ecology and Evolutionary Biology, Department of Biological and Environmental Science, University of Jyväskylä, Jyväskylä, Finland; 5 Life and Health Sciences Research Institute (ICVS), School of Medicine, University of Minho, Braga, Portugal

**Keywords:** African jerboas, Cryptic diversity, Demographic history, Deserts, *Jaculus*, Local adaptation, Phylogenetics, Reproductive isolation, Sahara-Sahel, Speciation

## Abstract

**Background:**

Climatic variation and geologic change both play significant roles in shaping species distributions, thus affecting their evolutionary history. In Sahara-Sahel, climatic oscillations shifted the desert extent during the Pliocene-Pleistocene interval, triggering the diversification of several species. Here, we investigated how these biogeographical and ecological events have shaped patterns of genetic diversity and divergence in African Jerboas, desert specialist rodents. We focused on two sister and cryptic species, *Jaculus jaculus* and *J. hirtipes*, where we (1) evaluated their genetic differentiation, (2) reconstructed their evolutionary and demographic history; (3) tested the level of gene flow between them, and (4) assessed their ecological niche divergence.

**Results:**

The analyses based on 231 individuals sampled throughout North Africa, 8 sequence fragments (one mitochondrial and seven single copy nuclear DNA, including two candidate genes for fur coloration: *MC1R* and *Agouti*), 6 microsatellite markers and ecological modelling revealed: (1) two distinct genetic lineages with overlapping distributions, in agreement with their classification as different species, *J. jaculus* and *J. hirtipes*, with (2) low levels of gene flow and strong species divergence, (3) high haplotypic diversity without evident geographic structure within species, and (4) a low level of large-scale ecological divergence between the two taxa, suggesting species micro-habitat specialization.

**Conclusions:**

Overall, our results suggest a speciation event that occurred during the Pliocene-Pleistocene transition. The contemporary distribution of genetic variation suggests ongoing population expansions. Despite the largely overlapping distributions at a macrogeographic scale, our genetic results suggest that the two species remain reproductively isolated, as only negligible levels of gene flow were observed. The overlapping ecological preferences at a macro-geographic scale and the ecological divergence at the micro-habitat scale suggest that local adaptation may have played a crucial role in the speciation process of these species.

## Background

Defining species and understanding the processes behind speciation are key components in studies of evolutionary ecology [1, 2]. It is suggested that divergent natural selection in contrasting habitats might trigger reproductive isolation through local adaptation, and consequently speciation, by limiting the chances of interaction between potentially reproducing individuals [3–5]. However, divergence between populations may be eroded by gene flow, particularly in the absence of evident barriers to dispersal [6, 7]. Despite the assumed oversimplification of the traditional categorization of speciation processes (allopatric, parapatric, and sympatric), the spatial context and the extent of gene flow between potentially diverging populations during speciation play a key role in determining whether, and how fast, reproductive isolation can evolve [8, 9]. Thus, the mechanisms of local adaptation and speciation are deeply influenced by the biogeographical and demographic history of populations, and may be triggered during periods of major ecosystem fluctuations [7, 10].

Northern Africa holds a great biogeographical interest owing to the strong species interactions (e.g., competition for limited and ephemeral resources), the wide diversity of habitats and heterogeneous landscapes, and the complex paleoclimatic and geological history [11–14]. Available phylogeographic studies in this region have uncovered substantial taxa diversification induced by the climate shifts that occurred during the Pliocene-Pleistocene interval (~ 5 million years ago [Mya]) and the successive range shifts of the Sahara desert [12–15]. These climatic fluctuations caused significant movements of the Sahara-Sahel boundaries, leading to alterations in the ecological composition of landscapes [11]. Such dynamics resulted in new selective pressures and/or geographic isolation within lineages, causing events of genetic diversification, adaptation, and eventually speciation [11].

As desert specialist rodent species, African Jerboas (*Jaculus* spp., Erxleben 1777, Dipodidae) have drawn the attention of researchers due to their broad distribution across the Saharan-Arabian region and their high phenotypic and genetic variation [16, 17]. Within the five recognized species in the genus, particular attention has been given to two putative sister cryptic species, until now considered as a single species due to incongruences between molecular and morphological studies [16–20]. These sister species present a broad and sympatric distribution throughout North Africa with overlapping phenotypic variation despite the putative divergent ecological preferences: the Lesser Egyptian Jerboa, *Jaculus jaculus* (Linnaeus 1758), characterized by a paler orangish dorsum with whitish-grey vibrissae associated with lighter sandy habitats; and the African Hammada Jerboa, *Jaculus hirtipes* (Lichtenstein 1823), described by a darker dorsum with grey vibrissae found mostly in darker rocky habitats [21] (Additional file 1: Figure S1). Over the years, the characterization of these species has not been consistent across studies. Some authors presented them as conspecific populations of the Lesser Egyptian Jerboa, a hypothesis widely acknowledged among taxonomists [18]. Studies relying on the genetic diversity of mitochondrial (*cytb* [16, 17, 19]) and nuclear DNA (*υWF* [17];) agree in distinguishing two divergent lineages corresponding to *J. jaculus* and *J. hirtipes*, with a broad and sympatric distribution in northwest Africa and report a high environmental and phenotypic overlap, including fur colour [17]. Moreover, Boratyński et al. [20], based on the phylogenetic and imaging analyses of the two species, reported a continuous within-species phenotypic variation in fur colour, making them almost indistinguishable in the field (Additional file 1: Figure S1a). The authors suggested that the two species persist genetically differentiated due to their ecological differences within the complex distribution patterns of sandy (lighter) and rocky (darker) habitats over North Africa [20] (Additional file 1: Figure S1b). However, a recent study, based on data collected from Israel and Sinai, claims that the two species can be distinguished in the field according to fur and tail coloration and morphology of male external genitalia and further confirms their different ecological requisites [22]. The observed controversy between studies suggests that the morphology of the two species may differ between regions, thus supporting the observed within-species phenotypic diversity in Boratyński et al. [20]. These conflicting results lead to a vast uncertainty of the current status of the two Jerboa species, where *J. hirtipes* is until now recognized as a subspecies of *J. jaculus*. It is, therefore, crucial to apply a more comprehensive approach to study this species complex to achieve a better understanding of the evolutionary history of these two forms, specifically, their level of genetic diversity, divergence, reproductive isolation, and ecological diversification.

Here, we assess the evolutionary history of the two putative species of African Jerboas by applying an integrative approach based on multi-locus genetic analyses and ecological niche tests. Our sampling encompasses all the North African range, thus covering the known distribution of these species [23], particularly focusing on individuals from West African regions, where both species overlap at the macrogeographic scale. Our main aims were: (1) to evaluate the phylogenetic divergence between species by analysing several independent markers (nuclear and mitochondrial) using species delimitation and species tree inference methods; (2) to estimate the divergence time and the demographic history of the two species; (3) to assess the levels of gene flow between species through estimations of the current genetic structure and levels of admixture, by analysing microsatellite data and isolation-with-migration (IM) models; and finally, (4) to give insights into the processes underlying speciation, taking into account niche overlap tests (i.e. addressing niche conservatism vs divergence), measures of gene flow, and past demography of the species. With this, we aim to deliver a more comprehensive view of this species complex and to clarify their taxonomic status. We hypothesize that if levels of gene flow are very low, they likely represent distinct species. Besides, we posit that our vast sampling and interdisciplinary approach will contribute to a better understanding of the evolutionary history and diversification processes of North African biota.

## Results

### Phylogenetic relationships and species delimitation in Jaculus spp.

**Fig. 1:**
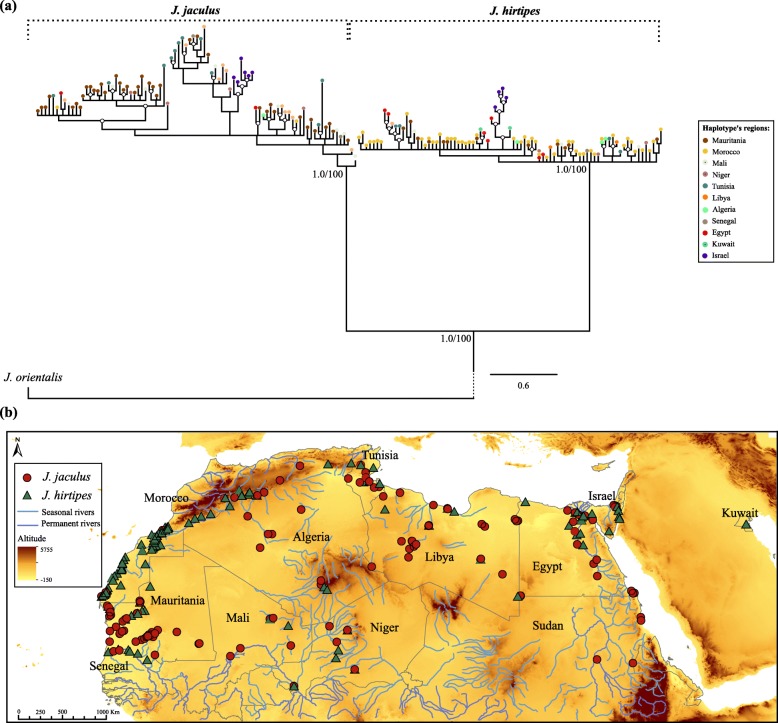
Phylogenetic relationship of *Jaculus* individuals and their geographic distribution across North Africa. **a** Phylogenetic tree based on Bayesian inference showing the relationship among the haplotypes of two *Jaculus* species for the *cytb* gene (*n* = 231; 170 haplotypes). Values on branches indicate Bayesian posterior probabilities support and bootstrap values of Maximum-Likelihood analysis, respectively. White circles indicate posterior probabilities and bootstrap values above 0.91/91, respectively, for internal nodes. On each clade, the respective species is indicated. *J. orientalis* (*n* = 7; 2 haplotypes) was used as outgroup. Each tip of the tree branches is coloured according to the country of origin of each individual belonging to a haplotype. **b** Geographical locations of all *Jaculus* individuals used in this study. Red (circles) and green (triangles) samples denote, respectively, *J. jaculus* and *J. hirtipes*

**Fig. 2:**
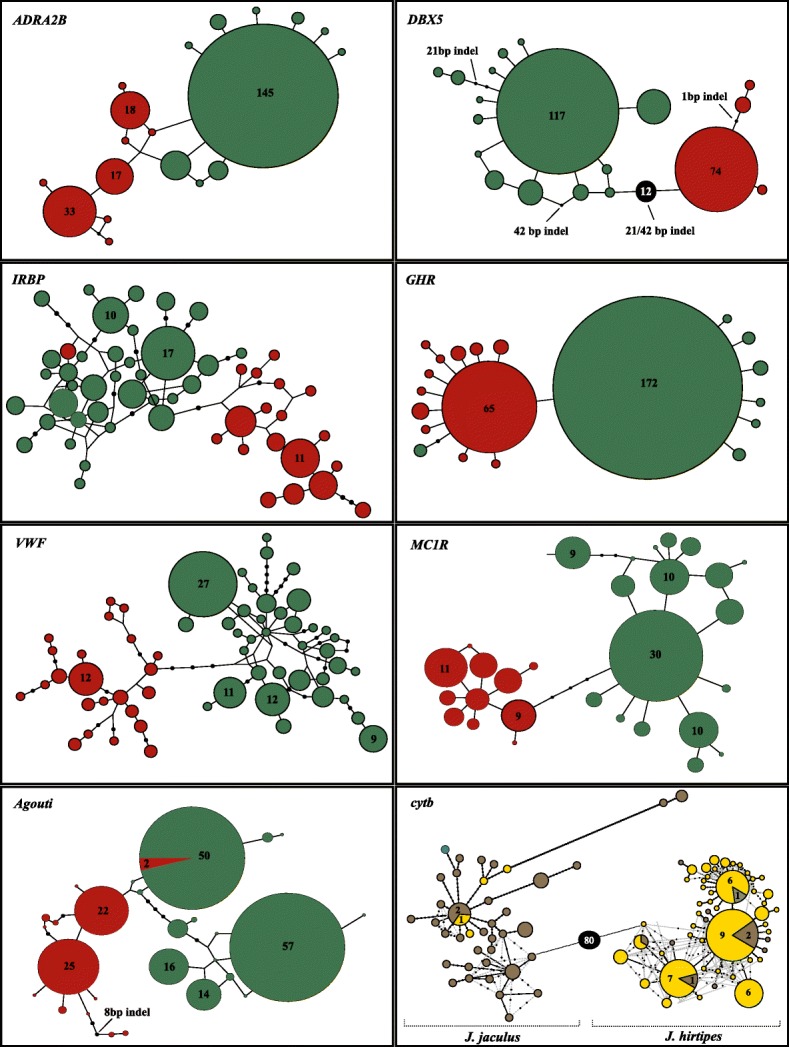
Statistical parsimony haplotype networks of *cytb*, X-chromosome intron (*DBX5*), and nuclear autosomal genes (*ADRA2B*, *IRBP*, *GHR*, *ƲWF, MC1R and Agouti*) of the *Jaculus* specimens successfully amplified with nuclear markers (*n* = 152 for *cytb*; the number of sequences used for each nuclear locus is specified in Table 2). Each circle represents one haplotype and the circle area is proportional to the frequency of each haplotype. Absolute frequencies are indicated for more common haplotypes. The size of the branches is proportional to the number of nucleotide differences between haplotypes, and dots on branches specify mutational steps where each node represents a single base difference. The insertion/deletion polymorphisms (indels) of *DBX5* and *Agouti* were coded as single mutations (see Additional file 1: Figure S1) and so the sizes of the indels are indicated on the respective mutational step. Due to the large number of mutational steps of *DBX5*, the number of mutational steps is indicated [12]. The same was performed for *cytb*. Haplotypes in the *cytb* network were coloured as in Fig. 1a to indicate that the field samples were collected in Mauritania, Morocco, Senegal, and Tunisia. The dashed lines represent the alternative relationships between haplotypes. Nuclear haplotypes are coloured according to the respective mitochondrial lineage: *J. jaculus* (in red) and *J. hirtipes* (in green) as in Fig. 1b

As the two species cannot be recognised in the field, samples were assigned to each of the species according to the two mitochondrial lineages previously described [17, 19, 20]. To do so, the mtDNA phylogeny was performed by combining the new collected specimens with data from previous studies ([17, 19, 20]; see Methods). This analysis recovered two main clades with high support, corresponding to the two putative species: *J. jaculus* and *J. hirtipes* (Fig. 1a). Both species hold a high number of haplotypes and high support values for the internal nodes within species (Fig. 1a). Within both species, distinct Israeli haplogroups are detected (Fig. 1a), suggesting some level of geographic isolation and genetic substructure in this region. In further analyses, individuals from these two mitochondrial lineages are classified as *J. jaculus* and *J. hirtipes*. The geographic distributions based on the mtDNA phylogeny of the two taxa overlap, thus confirming that *J. jaculus* and *J. hirtipes* persist in sympatry at a macrogeographic scale (Fig. 1b), as also observed in Fig. 2. The two species are also differentiated at nuclear loci, with nearly absent allele sharing (Fig. 2). For *GHR* locus, one individual from Bojador in the Atlantic coast of Morocco is homozygote for one allele that clustered within *J. jaculus*. This individual clustered within *J. hirtipes* at all other loci. In *IRBP* and *Agouti* genes the opposite pattern occurred: one individual from the Inchiri region in Western Mauritania had alleles from *J. hirtipes*, whereas it grouped with *J. jaculus* in the other loci analysed (Fig. 2).

**Fig. 3:**
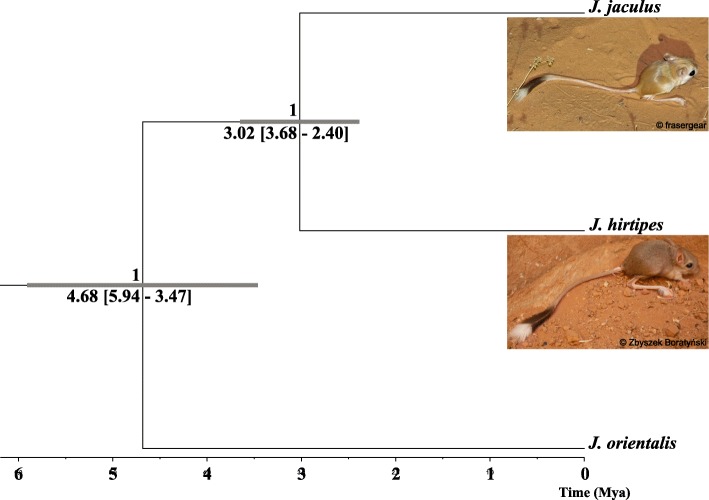
*BEAST species tree inference output for *cytb* and the seven single copy nuclear DNA loci analysed. The posterior probability of each split is shown on each node and grey bars display the 95% highest posterior density intervals for the estimated split times between the two lineages and *Jaculus* sp. – *J. orientalis,* by applying a *cytb* mutation rate of 0.176 (divergence estimates are presented below bars). Branch lengths are proportional to time according to the mutation rate used for *cytb*

Bayesian species delimitation consistently supports two species, *J. jaculus* and *J. hirtipes*, plus an outgroup species included in the analysis: *J. orientalis*, with the maximum posterior probability (speciation probability = 1). Moreover the probability of having three different species was 1 (P[3]=1), leaving P[2] and P[1] with 0. The species tree inferred by *BEAST recovered two strongly supported speciation events: an ancient split separating *J. orientalis*, and a more recent speciation node delimiting *J. jaculus* and *J. hirtipes* (Fig. 3). Calibration of the tree showed that the split between *J. orientalis* and the two other *Jaculus* species occurred along the Late Miocene-Pliocene transition, approximately 4.680 Mya (95% highest posterior density (HPD): 3.470–5.940 Mya). The split between *J. jaculus* and *J. hirtipes* is estimated to have occurred during the transition of Pliocene to Pleistocene, about 3.020 Mya (95% HPD: 2.400–3.680 Mya).

### Assessing the levels of gene flow

Levels of gene flow were assessed through Isolation-with-Migration (IM) models [24–26]. Estimations of the effective population sizes detected slightly higher values for *J. jaculus* (maximum-likelihood estimates and respective 95% posterior density intervals: 6.082 [4.763–7.463] millions) than for *J. hirtipes* (5.619 [4.478–6.742] millions), with an ancestral population size of 5.619 (0.967–9.558) millions. The divergence time between the putative species is estimated to be about 3.395 (1.867–5.482) Mya. Population migration rates were found to be significant in log-likelihood-ratio (LLR) tests [27], wherein a higher proportion of migrants per generation was detected from *J. jaculus* to *J. hirtipes* (0.133 [0.027–0.253] than from *J. hirtipes* to *J. jaculus*: 0.077 [0.005–0.163], *p* < 0.001). The posterior densities for all parameters were consistent across independent runs. Analyses were also performed without the two candidate genes for fur coloration, *MC1R* and *Agouti*, in order to assess potential bias towards putatively selected loci and results showed similar estimates (see Additional file 1: Table S1).

### Population genetics and demographic history

Population genetic divergence was high for *cytb* gene between *J. jaculus* and *J. hirtipes* (10.00%), but slightly lower than that observed between both species and the outgroup (*J. orientalis*; 12.00%). The *DBX* intron also revealed a high divergence between *J. jaculus* and *J. hirtipes* (3.00%), even higher than the genetic divergence separating *J. orientalis* and *J. jaculus* (0.40%), but similar to the genetic divergence between *J. hirtipes* and *J. orientalis* (3.30%). Divergence found in the autosomal loci was generally lower but among these, the *Agouti and υWF* genes presented the highest divergence (Table 1).

**Table 1:** Average genetic divergence (Dxy) and net nucleotide divergence (Da) between *J. jaculus and J. hirtipes,* between *J. jaculus-J. hirtipes* and *J. orientalis*, and other related rodent species

Locus	*J. jaculus/ J. hirtipes*	*J.orientalis/ J. jaculus*	*J.orientalis/ J. hirtipes*	*J.orientalis/ Jaculus spp.*	Other rodents
*Cytb*					
Dxy	10.00 (1.00)	12.00 (1.00)	12.00 (1.10)	12.00 (1.00)	12.50 (0.90)^b^ 7.80 (0.80)^c^
Da	8.80 (0.90)	10.80 (1.00)	11.40 (1.00)	9.00 (0.80)	–
*DBX5*^a^					
Dxy	3.00 (0.90)	0.40 (0.30)	3.30 (1.00)	2.40 (0.70)	1.10 (0.60)^d^
Da	3.00 (0.90)	0.30 (0.30)	3.30 (1.00)	1.70 (0.50)	–
*ADRA2B*					
Dxy	0.50 (0.20)	1.10 (0.30)	0.70 (0.20)	0.80 (0.20)	2.70 (0.50)^e^
Da	0.30 (0.20)	0.60 (0.20)	0.30 (0.20)	0.40 (0.20)	–
*GHR*					
Dxy	0.20 (0.10)	0.80 (0.20)	0.60 (0.20)	0.70 (0.20)	0.40 (0.20)^f^
Da	0.10 (0.10)	0.50 (0.20)	0.30 (0.20)	0.30 (0.20)	–
*IRBP*					
Dxy	0.80 (0.20)	0.80 (0.20)	0.60 (0.20)	0.70 (0.20)	0.50 (0.20)^g^
Da	0.40 (0.10)	0.60 (0.20)	0.40 (0.20)	0.40 (0.10)	–
*ʋWF*^a^					
Dxy	1.30 (0.30)	3.00 (0.50)	3.30 (0.50)	3.30 (0.50)	1.40 (0.30)^h^
Da	0.80 (0.20)	2.60 (0.50)	2.80 (0.50)	2.60 (0.50)	–
*Agouti*					
Dxy	1.90 (0.50)	1.60 (0.40)	1.60 (0.50)	1.80 (0.50)	0.70 (0.40)^i^
Da	0.90 (0.30)	1.30 (0.50)	1.20 (0.40)	1.00 (0.40)	–
*MC1R*					
Dxy	0.80 (0.20)	1.30 (0.40)	1.60 (0.40)	1.50 (0.40)	0.90 (0.30)^j^
Da	0.50 (0.20)	1.10 (0.30)	1.40 (0.40)	1.20 (0.30)	–

The standard errors (in parenthesis) are based on 10,000 bootstrap replicates of the p-parameter; all estimates are given as percentages

*Dxy* Average number of nucleotide substitutions per site between populations (average raw DNA divergence), *Da* Number of net nucleotide substitutions per site between populations (average net DNA divergence)

^a^Values obtained after removing the regions with significant within-locus recombination

^b^*Microtus arvalis* (GQ352469) / *Microtus agrestis* (GQ352470) [28]

^c^*Microtus arvalis* (AY513809) / *Microtus kirgisorum* (AY513809) ([29, 30]; respectively)

^d^*Microtus arvalis* (JX284377) / *Microtus agrestis* (JX284376) [31]

^e^*Acomys russatus* (FM162045) / *Acomys cahirinus* (FN984740) ([32, 33]; respectively)

^f^*Allactaga bullata* (JQ347909) / *Allactaga balikunica* (KM397227) ([34, 35]; respectively)

^g^*Allactaga bullata* (JQ347929) / *Allactaga balikunica* (KM397136) ([34, 35]; respectively)

^h^*Microtus agrestis* (FM200055) / *Microtus socialis* (FM162067) ([32, 36]; respectively)

^i^*Peromyscus polionotus* (DQ482897) / *Peromyscus maniculatus* (DQ482892) [37]

^j^*Peromyscus polionotus* (EU020068) / *Peromyscus maniculatus* (EU020066) [38]

The *cytb* gene displayed the highest intraspecific diversity, with higher values observed within *J. jaculus* than within *J. hirtipes* (Table 2). The *DBX5* intron exhibited the lowest diversity, and the autosomal genes, *IRBP*, *υWF* and *MC1R* had intermediate levels, with the highest diversity values observed for *J. hirtipes*, contrary to that observed in the mtDNA (Table 2). The *Agouti* gene also presented high levels of nucleotide diversity in *J. hirtipes* but not in *J. jaculus*. Compared with other autosomes, *GHR* recovered the lowest values of genetic diversity (Table 2). Overall, neutrality tests show negative values for almost all loci in the two species for Tajima’s D and Fu’s *F*_*s*_ statistics (Table 2).

**Table 2:** Diversity estimates within *Jaculus* species

Locus	Species	L	n	S	H	Hd(SD)	π (SD)%	*θ*_*W*_ (SD)%	*D*	*F*_*S*_	*R*_2_
*Cytb*	*J. jaculus*	897	96	156	87	0.997 (0.002)	1.61 (0.09)	3.39 (0.86)	−1.84^**^	−89.13^***^	0.04^**^
	*J. hirtipes*	897	137	102	83	0.980 (0.005)	0.58 (0.03)	2.07 (0.51)	−2.35^***^	− 107.96^***^	0.02^***^
*DBX5*	*J. jaculus*	311	84	3	4	0.220 (0.059)	0.09 (0.03)	0.20 (0.10)	−1.02	−1.96	0.04
	*J. hirtipes*	306	180	7	8	0.208 (0.040)	0.07 (0.01)	0.40 (0.20)	−1.77^*^	−8.94^***^	0.02
*DBX5 non-r*ec	*J. jaculus*	301	84	2	3	0.217 (0.057)	0.07 (0.02)	0.10 (0.10)	−0.71	−0.95	0.06
	*J. hirtipes*	296	171	6	7	0.166 (0.038)	0.06 (0.01)	0.40 (0.20)	−1.73	−8.21^***^	0.02
*ADRA2B*	*J. jaculus*	693	72	7	9	0.705 (0.031)	0.30 (0.02)	0.20 (0.20)	0.32	−0.87	0.13
	*J. hirtipes*	693	180	11	11	0.345 (0.045)	0.06 (0.01)	0.30 (0.10)	−1.85^*^	−9.67^*^	0.02
*GHR*	*J. jaculus*	798	80	10	11	0.378 (0.070)	0.05 (0.01)	0.20 (0.10)	−2.09^*^	−12.09^**^	0.03^*^
	*J. hirtipes*	798	183	11	10	0.147 (0.036)	0.03 (0.008)	0.20 (0.09)	−2.12^*^	−12.93^***^	0.02^*^
*IRBP*	*J. jaculus*	1058	50	25	19	0.905 (0.023)	0.35 (0.05)	0.53 (0.20)	−1.07	−6.48	0.07^*^
	*J. hirtipes*	1058	106	23	35	0.948 (0.010)	0.39 (0.002)	0.41 (0.13)	−0.19	−19.37	0.09
*ʋWF*	*J. jaculus*	873	40	31	23	0.938 (0.026)	0.57 (0.07)	0.83 (0.28)	−1.11	−10.77	0.08^*^
	*J. hirtipes*	874	132	33	37	0.933 (0.012)	0.56 (0.02)	0.69 (0.20)	−0.70	−15.72	0.07
*ʋWF non-*rec	*J. jaculus*	516	26	6	6	0.717 (0.079)	0.38 (0.05)	0.30 (0.15)	0.76	0.14	0.16
	*J. hirtipes*	514	127	18	14	0.861 (0.016)	0.44 (0.27)	0.65 (0.21)	−0.89	−2.48	0.06
*Agouti*	*J. jaculus*	453	66	14	12	0.746 (0.037)	0.39 (0.05)	0.65 (0.24)	−0.06	− 0.06^*^	0.10^***^
	*J. hirtipes*	461	162	14	15	0.765 (0.021)	1.07 (0.02)	0.54 (0.18)	−0.09^*^	−0.36	0.08^***^
*MC1R*	*J. jaculus*	894	56	9	11	0.890 (0.016)	0.19 (0.01)	0.22 (0.09)	−0.06	−0.08	0.11
	*J. hirtipes*	894	110	13	19	0.890 (0.019)	0.25 (0.02)	0.28 (0.10)	−0.06	−0.21^*^	0.09

In loci with significant levels of within-locus recombination, values are shown for the recombinant and non-recombinant datasets (denoted as “non-rec’”)

*L* number of sites excluding gaps, *n* number of sequences, *S* number of segregating sites, *H* number of haplotypes, *Hd* haplotype diversity, *π* nucleotide diversity per site, *θ*_*W*_ computed from the number of segregating sites, *D* Tajima’s D, *F*_*S*_ Fu’s *F*_*S*_, *R*_2_ Ramos-Onsins & Rozas’s *R*_2_

Significant values indicated ^*^(*P* < 0.05), ^**^(*P* < 0.01), ^***^(*P* < 0.001)

**Fig. 4:**
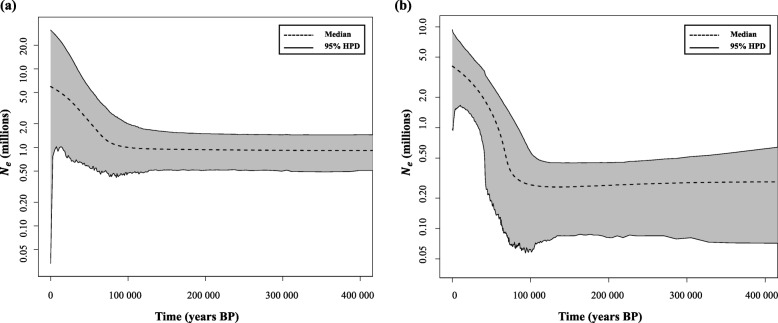
Extended Bayesian Skyline plots (EBSP) of the effective population size through time obtained from the three MCMC simulations for **a** *J. jaculus* and **b** *J. hirtipes*. Dashed black line is the median effective population size *N*_*e*_ in millions, multiplied by one (mean generation time in years). Solid black lines are the 95% highest posterior density limits. The y-axis is displayed on a log scale for simplicity

Estimated effective population sizes through time revealed signs of expansion in both *J. jaculus* and *J. hirtipes*, which may have started around 100,000 years ago (Fig. 4). The analysis suggests that the demographic expansion may have started approximately at the same time in the two species. Estimations of contemporary population sizes show relatively higher estimates for *J. jaculus* (~ 9 and ~ 5 millions in *J. jaculus* and *J. hirtipes* respectively, Fig. 4), although with higher confidence intervals.

### Population structure and admixture

**Fig. 5:**
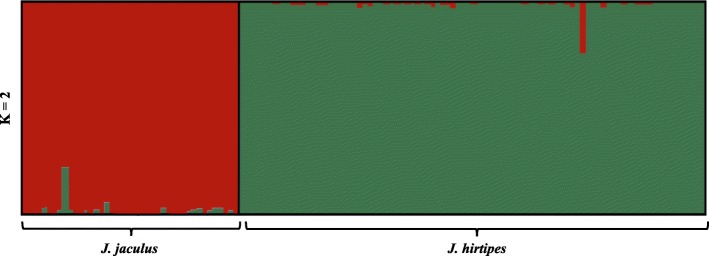
Structure bar plot of Bayesian assignments of individual to the respective cluster (K = 2). Vertical bars indicate individuals and the colours within each bar correspond to the probability of membership of each specimen to a cluster (in red – *J. jaculu*s; in green – *J. hirtipes*)

Six loci (Jac04, Jac07, Jac11, Jac12, Jac24, and Jac27), out of the 13 microsatellites initially tested, revealed significant deviations from the Hardy-Weinberg equilibrium, presenting heterozygote deficiency (Additional file 1: Table S2). Moreover, one locus (Jac01) amplified only samples belonging to *J. jaculus*. After removing these markers, assessments of population structure were performed with the six remaining loci for a total of 132 specimens (40 and 92 for *J. jaculus* and *J. hirtipes*, respectively). Structure Harvester [39] results highlighted K = 2 as the most likely number of clusters best explaining the variation in our dataset (for both DeltaK and L(K) methods, see Additional file 1: Table S3). Structure bar plot exhibited a clear separation between the two species (Fig. 5). Additional intraspecific substructure was identified within *J. hirtipes* for *K* = 3 (Additional file 1: Figure S2a), although with no evident geographical structure (Additional file 1: Figure S2b). The Principal Coordinate Analysis showed that PC1 (16.53%) and PC2 (5.30%) separate individuals between and within species, respectively (Additional file 1: Figure S3). The observed low intraspecific substructure could reflect the lack of power of the markers used. High levels of polymorphism were detected both for the whole dataset (i.e. two species as a single group) and within species, with similar allelic diversity observed between species for all microsatellite markers, varying from 9 to 29 alleles, although with a higher heterozygosity observed in *J. jaculus* (Table 3). Estimates of the F-statistics show significant differentiation (*F*_*ST*_) between species (Table 3).

**Table 3:** Mean heterozygosity (observed and expected) and F-statistics for *J. jaculus* and *J. hirtipes* based on microsatellite loci

Cluster	N	Na	Ne	Ho	He	uHe	F_IS_	F_IT_	F_ST_
*J. jaculus*									
Mean (SE)	42	12.83 (1.38)	5.74 (0.81)	0.80 (0.03)	0.81 (0.03)	0.82 (0.03)	–	–	–
*J. hirtipes*									
Mean (SE)	90	12.83 (2.44)	5.86 (1.39)	0.69 (0.13)	0.70 (0.14)	0.71 (0.14)	–	–	–
Total									
Mean (SE)	132	12.83 (1.34)	5.80 (0.78)	0.75 (0.07)	0.76 (0.07)	0.76 (0.07)	0.02 (0.01)	0.18^*^ (0.03)	0.16^*^ (0.03)

*SE* Standard error, *N* Sample Size, *Na* Number of Alleles, *Ne* Number of Effective Alleles, *Ho* Observed Heterozygosity, *He* Expected Heterozygosity, *Uhe* Unbiased Expected Heterozygosity, *F*_*IS*_ Inbreeding Coefficient, *F*_*IT*_ Total Fixation Index, *F*_*ST*_ Fixation Index

Significant values indicated ^*^(*P* < 0.5)

### Niche overlap

Overall, the observed niche overlap (Schoener’s D) for both habitat and topo-climatic variables, is high (D > 0.4) at the 5 × 5 km scale, and for topo-climatic factors at the 1 × 1 km scale (Additional file 1: Figure S4). However, niche overlap for habitat measured in the 1 × 1 km scale was relatively low (D = 0.25). Niches were detected not to be equivalent (i.e. niches not constant when randomly reallocating individuals between the two species’ ranges) since equivalency tests were significant in all cases (*p* < 0.05) (Additional file 1: Figure S4). Similarity tests were also significant (*p* < 0.05) and the value of D (in red, Additional file 1: Figure S4) is placed in the second tail of the distribution, therefore, the species tend to have similar patterns of topo-climate and habitat selection, more than expected by chance.

## Discussion

### Two closely-related species: the African Hammada Jerboa and the Lesser Egyptian Jerboa

Our comprehensive approach clarified the phylogenetic relationship between the two jerboa species, with widespread and overlapping distributions across North Africa (Fig. 1). The phylogenetic inferences of mitochondrial DNA revealed two well defined and strongly supported clades (Fig. 1a), as shown in previous studies [17, 19, 20, 22]. Moreover, we showed for the first time that the two mtDNA lineages can be further distinguished by seven single copy nuclear markers (Fig. 2) and six microsatellite loci (Fig. 5). By applying the coalescent methods of species delimitation and species tree inference [40], two well delimited clades with fully resolved nodes can be observed (Fig. 3). Hence, we have revealed that the loci analysed both at nuclear and mitochondrial DNA agree in the identification of two different species.

Average *cytb* nucleotide divergence (10.0%) was slightly lower to previously documented for these species (10.5% [19]; and 10.6% [17]), but beyond intraspecific variation usually observed in rodents (average 2.1%, up to 6.29 [16, 31, 41]). Moreover, the observed divergence is slightly above the average genetic distance observed between sister rodent species (average: 9.6%, range 2.7–19.2 [41, 42]). In particular, the divergence between the two jerboas were considerably higher than between closely-related *Microtus* species: *M. arvalis* and *M. kirgisorum* (7.8%), but lower than between distant taxa: *M. arvalis* and *M. agrestis* (12.5%; Table 1) [30, 33]. For nuclear loci, the genetic divergence observed between *J. jaculus* and *J. hirtipes* in the *IRBP*, *DBX5* and *Agouti* genes was higher than that observed between other closely-related rodent species, while for *ADRA2B* the values were considerably lower (Table 1). The remaining autosomal genes had similar values of genetic divergence (Table 1). Overall, the observed genetic divergences between *J. jaculus* and *J. hirtipes* are compatible with their classification as two different species.

### Insights into the evolutionary history of Jaculus species

Our species tree inference estimates a divergence time between *J. orientalis* and *J. jaculus-J. hirtipes* during the Late Miocene-Pliocene transition, around 4.680 (3.470–5.940) Mya (Fig. 3). These results are in the range of previous estimates of divergence time between *J. orientalis* and other *Jaculus* species (5.97 [5.29–7.09] Mya [35]). The split between *J. jaculus* and *J. hirtipes* is estimated to be along the Pliocene-Pleistocene boundary, around 3.395 (1.867–5.482) Mya according to IM and around 3.020 (2.400–3.680) Mya based on *Beast (Fig. 3). Although these estimates indicate an older divergence of *Jaculus* species when compared to other rodent species such as *Acomys* (1.25 [0.65–1.94] Mya [43]) or *Mastomys* (2.82 [1.61–4.20] Mya [44]), this should be interpreted with caution due to the lack of accurate substitution rates in these rodent groups, and the unavailability of dated fossil records to time-calibrate the phylogeny. Nonetheless, according to the dated estimates, the divergence between these two species coincided with climatic fluctuations across North Africa. Previous studies showed that recurrent humid climatic phases (the so-called “green” Sahara) counteract expansion events of xeric species, like jerboas, constraining species ranges to geographically isolated populations [11].

Previous assessments of the historical demography of *Jaculus* species indicated potential signs of expansions in both species [17]. Our results corroborate these findings and suggest similar times of population expansion for *J. jaculus* and *J. hirtipes*, although with slightly different effective population sizes (Fig. 4). Neutrality tests and reconstructions of population dynamics for each of the species rejected a demographic model of population at equilibrium (Table 2), and indicated signs of population expansion (Fig. 4). This could have started around 100,000 years ago, coinciding with the major climatic oscillations of the Upper Pleistocene of North Africa that induced critical changes in the genetic signature of several vertebrate species, including other West African rodents [43, 45–48]. However, we cannot exclude that this pattern of population expansion results from our sampling based on the collection of single individuals from different locations rather than entire populations. This could have increased the number of rare alleles, artificially resembling a pattern of demographic expansion. Future studies focused on the analyses of populations should allow to distinguish between these two different hypotheses.

### Assessing gene flow between J. hirtipes and J. jaculus

*Jaculus jaculus* and *J. hirtipes,* are often found in sympatry in North Africa, thus increasing the probability of hybridization. Two out of 152 analysed individuals presented alleles at two nuclear markers that are typical from the other species, which could result from incomplete lineage sorting or introgression. However, the IM analysis supported gene flow between the two species in both directions, although higher towards *J. hirtipes*. The microsatellite data further suggests potential admixture among species (Fig. 5), although the majority of the individuals also revealed a high membership probability to the respective species (Fig. 5). Despite being significant, the IM estimated levels of gene flow were very low, suggesting that the level of isolation between species might be very high. Moreover, these estimates (*2 Nm* of 0.077 into *J.jaculus* and 0.133 into *J. hirtipes*) were lower than those usually reported between subspecies of mammals, where *2 Nm* values can go up to 1.50 (e.g., [49, 50]). Our findings, therefore, show that, despite gene flow, *J. jaculus* and *J. hirtipes* remain strongly genetically differentiated, suggesting strong reproductive isolation.

### What drives speciation in this system?

Population divergence in the presence of gene flow often suggests that local adaptation is a crucial driver of differentiation between two or more populations [51–53]. Persistent habitat-phenotype covariation within jerboas (and other desert rodents) suggests that natural selection may be the trigger of phenotypic divergence [20, 54]. Indeed, previous studies have suggested that, despite the coexistence of the two jerboa species in sympatry across much of Sahara-Sahel, they might segregate into distinct micro-habitats, perhaps in response to strong predator driven selection [17, 20]. These species might, therefore, persist in different micro-habitats associated with the admixture of sandy (lighter) and rocky (darker) micro-habitats over North Africa, where *J. jaculus* and *J. hirtipes* are more frequent, respectively [20]. A tidier micro-habitat preference was previously suggested for *J. jaculus*, implying that *J. hirtipes* may be competitively excluded to suboptimal micro-habitats, which could explain its slightly lower effective population size. We found a strong niche overlap between species and similar patterns of habitat selection (Additional file 1: Figure S4). This might explain the observed overlapping distribution in fur colour variation in both species (Additional file 1: Figure S1a). However, when tests are performed at a local scale (i.e. 1 × 1 km), the habitat component of the niches has lower overlap (Additional file 1: Figure S4), thus suggesting that the two species might persist in ecological separation at a micro-habitat scale. It is thus possible that the observed divergence between species might have arisen through ecological adaptation at the micro-scale (lower than 1 km), a pattern also observed in other organisms (e.g. marine sea snails [55]). Nonetheless, the genetic divergence between the two lineages suggests that this could have happened during a period of geographical isolation. More studies are, therefore, needed to fully disentangle these and other putative scenarios. Finally, mating preference experiments are required to test if fur colour is a determinant factor for their mating preferences, which would help to clarify the main drivers of reproductive isolation between the two species.

## Conclusions

Our comprehensive analyses, based on both mitochondrial and nuclear DNA, provide evidence for two different species of African Jerboas that have a similar distribution across North Africa: *J. jaculus* and *J. hirtipes*. Our results propose that these two species might have experienced demographic expansions since the Late Pleistocene period, with a higher effective population size observed for *J. jaculus*. Despite the detection of small levels of gene flow between species, the two species persist strongly differentiated. Moreover, analysis of niche divergence suggests that *J. jaculus* and *J. hirtipes* are ecologically separated at a micro-habitat scale. These findings suggest that natural selection at a micro-scale could have driven the speciation process. However, the divergence at multiple loci also suggest that this could have involved some geographic isolation. Further analyses to assess levels of introgression and to identify loci involved in adaptation across the genome are thus required to fully understand the processes driving the observed diversification of North African jerboas.

## Methods

### Sampling and DNA extraction

A total of 231 samples distributed throughout North Africa, including 152 tissue samples collected in the field and 79 samples obtained from museum collections, were used in this study (Additional file 1: Table S4 and Fig. 1). Tissue samples were collected from road-killed (*n* = 126) and live trapped animals (*n* = 26) during several field expeditions in North-West Africa or received from collaborators between November 2011 and February 2015 ([54, 56, 57]; Additional file 1: Table S4). From the 26 live-captured animals, 14 were anesthetised using a recommended dosage of isoflurane followed by cervical dislocation for euthanasia [56]. Specimens were preserved at the Natural History Museum of the Département de Zoologie et Ecologie Animale, Institut Scientifique de Rabat, Morocco. For the other 12 animals, only ear tissue samples were collected. All methods were performed in accordance with the relevant guidelines and regulations (see Ethics approval and consent to participate section). Tissue samples were preserved in 96% ethanol for genetic analyses at the moment of collection. A total of 54 samples were already used in previous studies, for *cytb* (51 samples) and *ʋWF* (21 samples) [17, 20]; Additional file 1: Table S4), but their genomic DNA was re-extracted and analysed for all markers used in this study. Additionally, 10 samples of *J. orientalis* were extracted and included as an outgroup species (Additional file 1: Table S4). Extractions of the genomic DNA from tissue samples were performed using EasySpin Kit, following the “Genomic DNA Minipreps Tissue Kit” protocol. Extractions of museum samples were performed in a separate and autonomous facility, under sterile conditions, using the QIAamp® DNA Micro Kit (QIAGEN), following the “Isolation of Total DNA from Nail Clippings and Hair” protocol. Extracted DNA was stored at − 20 °C.

### DNA amplification and sequencing

One mitochondrial locus (cytochrome *b*, *cytb*, 897 bp) and seven nuclear loci were amplified, including two candidate genes for colour morph variation (the complete coding region of the melanocortin 1 receptor, *MC1R*; and a fragment of the exon 2 of the *Agouti* gene and part of an intron), one X-linked gene (intron 5 from the developing brain, homeobox gene, *DBX*) and four autosomal genes (exon 10 from the growth hormone receptor, *GHR*; exon 1 from alpha-2B adrenergic receptor, *ADRA2B*; exon 1 from the interstitial retinoid binding protein, *IRBP*; and exon 28 from the ʋon Willebrand factor, *ƲWF*), producing a total of 5369 bp. Partial amplification of the *cytb* gene (897 bp) was performed for the entire set of samples (231 samples, contemporaneous and museum) using two primer pairs previously designed for *Jaculus* species (Jac1Fw, Jac1Rv, Jac4Fw, Jac4Rv [17]). The reconstruction of the DNA fragment for the museum samples was done in several steps to produce overlapping sequences in order to obtain the entire fragment. In some cases, only a short fragment (325 bp) of the gene was amplified, which was obtained combining two primers, Jack4Fw and Jack1Rv (primers, references and PCR conditions for *cytb* are described in Additional file 1: Table S5). As the amplification of the short fragment was accomplished for a larger number of samples, this was used to confirm the phylogeny with the long fragment. Nuclear loci and microsatellites were amplified only on samples collected during field work (152 samples; Additional file 1: Table S4). PCR products of both mitochondrial and nuclear genes were purified with a commercial kit (Qiagen) and both strands were sequenced on an ABI 3130xl Genetic Analyser (AB Applied Biosystems). For the autosomal genes, sequencing of both strands was performed in an external laboratory (Macrogen Inc.). Additionally, available sequence data for the *cytb* gene of our target species (164 sequences) were downloaded from GenBank and included in the analyses (Additional file 1: Table S6).

### Sequence alignment and phylogenetic analyses

Each sequence was first verified and manually aligned using SEQSCAPE v2.6 [58]. Alignments for each locus were then refined with CLUSTAL W [59] implemented in ClustalX v2.0 [60] and edited manually in BIOEDIT v7.1.3 [61] in order to minimize the number of base pairs in the alignment spanned by insertion/deletions (indels). Polymorphic positions for each sequence from nuclear loci were carefully examined to ensure precise and consistent identification of double peaks in heterozygotes. Heterozygous sequences for indels were resolved manually from offset chromatogram peaks, combing the reverse and forward sequences [62]. Nuclear haplotypes were inferred using PHASE v2.1 [63, 64] with three runs performed for each locus with 10,000 burn-in steps and 10,000 interactions. Input files were created in SEQPHASE [65]. Phased heterozygotes holding indels were included in SEQPHASE as “known haplotype pairs”. Haplotypes presenting probability phase calls below 80% were discarded from the analysis to ensure that only reliable haplotypes were used in downstream analyses. The indels observed in the *DBX* (21 and 42 bp; Additional file 1: Figure S5) and in the partial *Agouti* gene (8 bp) were coded manually and were included in network reconstruction but excluded in further analyses due to their large sizes. Haplotypes for the *cytb* gene were inferred with DnaSP v5 [66].

Phylogenetic analyses were performed for the *cytb* locus. The Akaike information criterion (AIC [67]) was used to select the best-fit model of sequence evolution for each locus alignment among the 88 available in the software jModelTest v2.1.4 ([68], Additional file 1: Table S7). The phylogenetic relationships between haplotypes were inferred by the Maximum-Likelihood (ML) approach in PHYML v3.0 [69] and the Bayesian phylogenetic inference (BI) implemented in MrBayes v3.2.0 [70]. ML analyses were performed with 1000 bootstrap pseudo replicates. Bayesian posterior probabilities were assessed from two runs with four chains of 1 million generations for the nuclear genes and 50 million generations for *cytb*, with a sampling frequency that provided a total of 10,000 samples for each run, discarding 25% of them as burn-in. Tracer v1.5 [71] was used to evaluate the convergence of the ESS values (effective sample size) for each analysis (ESS > 500). Resulting trees were drawn with FIGTREE v1.3.1 [72].

Haplotype networks were generated for each nuclear gene individually using parsimony calculations in TCS v1.21 [73] considering gaps as a fifth state. Each indel of the *DBX5* and *Agouti* locus was considered as a single mutational step, regardless of the corresponding size (Fig. 2). Analyses were performed for each locus with a connection limit of 95%. *DBX* locus presented disconnected haplotypes and so networks were redrawn with the connection limit fixed at 90% in order to link the more unrelated groups and see the number of mutational steps among them. Networks were edited using tcsBU [74]. The *cytb* haplotype network was performed with the R packages “pegas” [75] and “ape” [76].

### Species delimitation and species tree inference

Alignments were first tested for the presence of within-locus recombination with SPLITSTREE v4.13.1 [77] and were found to be significant in regions of the *DBX5* and *υWF* genes. These were further analysed with IMgc [78] to reduce the dataset to the largest non-recombinant blocks. Moreover, in order to validate the assignment of individuals to the two previously described mitochondrial lineages [16, 17, 19, 20, 22], the program Bayesian Phylogenetics and Phylogeography (BP&P) v3.1 was used to assess the status of species delimitation. Our analyses included the mtDNA and the seven single copy nuclear DNA regions. Due to the large sample size of our dataset, only 30 individuals, chosen randomly, were analysed for each lineage on each locus. The same outgroup sequences of *J. orientalis* were used for this analysis. Population size parameters (θ) and divergence time at the root of the species tree (τ) were estimated with the gamma prior G(2, 1000), while the Dirichlet prior was assigned to all other divergence time parameters. We used “algorithm 0” with the fine-tune parameter set to default. Each species delimitation model was assigned equal prior probability. For the MCMC, samples were collected for 1,000,000 generations, with a sampling interval of 2 and a burn-in of 10%. Each analysis was run 3 times to confirm consistency among runs.

The same dataset was also used to infer the species tree by applying the multispecies coalescent model implemented in *BEAST [40], part of the BEAST v2.3.0 package [79]. Samples were assigned according to the two mitochondrial lineages defined above. The input file was produced with the application BEAUti v2.3.0, also included in the BEAST package. Preliminary analyses were carried out to evaluate which clock-like evolution model best fits the data by comparing a relaxed with a strict molecular clock. Based on these trial runs the final analysis was accomplished with an uncorrelated lognormal relaxed clock, using the HKY + I + G substitution model for *cytb*. Analyses of the nuclear loci were carried out with an HKY (+I for *ƲWF*, *ADRA2B*, *IRBP*, *MC1R* and *Agouti*) substitution model under a strict molecular clock (Additional file 1: Table S5).

Times of divergence were estimated using *cytb* as the reference gene. A fossil-based calibration of substitution rates was not possible due to the poor fossil record of *Jaculus* in North Africa. Similarly, the well-known calibration point Muridae-Rodentia was not used due to the likely saturation effect associated with the ancientness of the divergence between Muridae and Dipodidae. Instead, we used the average *cytb* substitution rate estimated for rodent species (0.176 substitutions/site/Myr [80]). Following these assumptions, the prior of the relaxed clock standard deviation was set to a normal distribution with a mean of 0.176 with *sigma* fixed at 0.05. This mutation rate was used in all subsequent analyses. The coalescent constant population size was used as tree prior and all the remaining priors were set to default. Three independent runs of 500 million generations were implemented, sampling trees and parameter estimators every 50,000 generations for all loci. The convergence of the runs was verified after the removal of a 10% burn-in using TRACER v1.5. Visual inspection of trace plots indicated a good sampling of all parameters for each *BEAST independent runs, with effective population sizes (ESS) above 1000, suggesting a good convergence of all parameters. The results from all runs were combined with LogCombiner v2.3.0, and the subsequent maximum clade credibility summary trees with posterior probabilities for each node were generated with TreeAnnotater v2.3.0 from the BEAST package. All the trees were visualized and edited with FIGTREE v1.3.1.

### Isolation-with-migration analyses

Species tree inferences performed with *BEAST incorporate the uncertainty associated with the coalescent process while estimating the phylogeny. However, it does not assume the possibility of occurrence of gene flow after the initial split. Thus, models of isolation-with-migration (IM) [27] implemented in the IMa2 software [24–26] were applied to infer whether gene flow has occurred between the two putative species. This method estimates the multi-locus effective population sizes (for present and ancestral populations), divergence times and migration rates under a model of isolation with migration [25, 27]. Analyses were performed with the mtDNA and the seven single copy nuclear DNA, and considering the two *Jaculus* species as populations. After several preliminary runs, two independent runs with different starting seeds were performed by sampling 200,000 genealogies per locus with 10% burn-in. Chain convergence was assessed by inspecting ESS values (ESS > 500) and by checking trend plots to verify whether each parameter had a normal distribution. We used a geometric model with the first heating term (ha) set to 1.05 and the second (hb) to 0.95 sampling through 80 chains (hn). Priors for population size, migration rates and splitting times were set to 15, 0.5, and 15, respectively, after assessing the convergence of runs in preliminary analyses. The HKY mutation model was applied to all loci and the same substitution rate as in *BEAST was specified to *cytb* (here scaled by the length of the locus [897 bp]: 1.96e-04, ranging from 1.40e-04 to 2.52e-04) in order to obtain the results in demographic units, considering 1 year of generation time [80]. Moreover, the log-likelihood ratio test (LLR) described by Nielsen and Wakeley [27] was used to assess whether migration rates were significantly different from zero, sampling over 400,000 trees, as implemented in the Load-Genealogy mode (L-mode) of IMa2.

### Population genetics and demographic analyses

Total (Dxy) and net (Da) divergences between lineages were calculated using p-distance parameter in MEGA v5.1. Additionally, the divergence among several related rodent species, based on published data, was inferred for comparison analysis [28–38]. Standard deviations for these divergences were estimated from 10,000 bootstrap replications. Nucleotide diversity (π), theta computed from the number of segregating sites (θ_*W*_), and haplotype diversity (Hd) were calculated per lineage for each locus analysed. Three test statistics, Tajima’s *D* [81], Fu’s *Fs* [82] and R_2_ [83] were performed to investigate deviations from neutral expectations, which could imply recent population expansion and/or signatures of selection. Significance was evaluated through 10,000 coalescent simulations. These statistics were assessed per locus for each lineage in DnaSP v5. Calculations were made separately for the entire data set and for the non-recombinant portions obtained with IMgc.

The dynamics of effective population sizes through time of the two lineages of *Jaculus* sp. were inferred with Extended Bayesian Skyline Plots (EBSP [84]), using a linear model in BEAST v2.3.0 and inputted through BEAUti v2.3.0. The same non-recombinant dataset used for species tree inference was analysed. The evolutionary models for each locus of each lineage were estimated in jModelTest v2.1.4, which resulted in similar models to the ones previously obtained (Additional file 1: Table S7). After preliminary analyses the evolutionary rates of the mitochondrial and nuclear loci were set to a strict molecular clock. The prior for the mean distribution of population sizes was optimized according to the population sizes estimated in preliminary runs, where different population size models were compared (Gamma, uniform, and exponential distributions) based on the ESS values, and was set with a coalescent prior and a constant population size [84]. Remaining priors were set as default. The MCMC parameters were the same as applied in *BEAST analysis. TRACER v1.5 was used to assess the convergence of the independent runs (ESS > 500). Results of the separate runs were combined with LogCombiner v2.3.0, part of the BEAST package, after discarding 10% as burn-in.

### Microsatellite selection and optimization

Since there were no specific microsatellite markers available for *Jaculus* spp. or closely related species, a microsatellite library was developed through high-throughput genomic sequencing (454 pyrosequencing) at GenoScreen (http://www.genoscreen.fr/en/) using *J. jaculus* individuals from distinct regions in North Africa. Detailed description of the optimization procedure can be found in Additional file 1. After optimization we used two multiplexes amplifying seven and four markers each, as well as two additional loci that had to be amplified individually in separate PCR reactions (Additional file 1: Table S8).

### Microsatellite genotyping

A total of 148 contemporary samples were genotyped for 13 microsatellite loci. Multiplex and individual reactions, primer concentrations and amplification conditions are summarized in Additional file 1. Allele data were obtained using GENEMAPPER v4.0 (Applied Biosystems 2006). Sizing bin windows were created manually and the automated scoring was checked by three independent observers to minimize genotyping errors. In order to assure consistency of results, 30% of the dataset was repeatedly genotyped in three independent runs. Inconsistent genotypes (~ 2% of all genotypes) were considered as missing data.

### Microsatellite analysis

As the sampling was continuous across the distribution and it is hard to delimit populations, these analyses were performed considering the two *Jaculus* species as two different populations. MICROCHECKER v2.2.3 [85] was used to assess the presence of genotyping errors due to null alleles and allele dropout. Linkage disequilibrium (LD) and deviations from Hardy-Weinberg Equilibrium (HWE) were estimated with GENEPOP on the Web (genepop.curtin.edu.au). The significance of the analysis were inferred according to the Bonferroni correction (0.05/[number of populations*number of loci]), and confirmed with three independent runs. Loci presenting significant deviations from HWE and from LD assumptions and with missing data above 40% were discarded from further analyses. Measures of genetic diversity and differentiation, such as allele frequencies, mean number of alleles sampled per locus and population and the corresponding allelic richness, observed (Ho) and expected (He) heterozygosity, and F-statistics were estimated with FSTAT v1.2 [86]. Individual-by-individual genetic distances that were used to compute a Principle Coordinate Analyses (PCoA) were calculated with GENALEX v6.0 [87]. The number of clusters and the quantification of admixture between lineages were inferred with the Bayesian Clustering software STRUCTURE v2.3.3 [88]. Analyses were accomplished by applying the admixture model with correlated allele frequencies. The software was run for the number of clusters (K) between 1 and 10 with 5 replicates of 1,000,000 MCMC iterations for each K value, following a burn-in period of 100,000 steps. Three independent analyses were performed to ensure similar posterior probabilities between runs. STRUCTURE HARVESTER v0.6.92 [39] was used to determine the probability of each K value. The most likely number of clusters (populations) was assessed using the mean values of likelihood [L(K)] and Delta K [89].

### Niche overlap

Resemblance of ecological niches between species was tested: for overlap using Schoener’s D Index (which ranges from 0, no overlap; to 1, total overlap), for niche equivalency (i.e. whether the niche overlap is constant when randomly reallocating the occurrences of both entities among the two ranges), and for niche similarity (i.e. whether the environmental niches are more similar than expected by chance [90]). The PCA-environment ordination approach developed by Broennimann et al. [91] was used for analyses. Tests were performed for two regions and scales, for the entire North Africa at ~ 5 × 5 km scale and for North-West Africa only (i.e. Mauritania and southern Morocco) at ~ 1 × 1 km scale, over two types of background data, composed by: (1) topo-climatic, including two topographic (altitude and slope) and 19 bioclimatic variables; and (2) habitat variables, including six Euclidian distances to habitat categories. Altitude and the 19 bioclimatic variables were downloaded from WorldClim (www.worldclim.org/bioclim). Slope was derived from a digital elevation model using the “slope” function from ArcGIS (ESRI 2011). Four of the habitat variables were constructed from land-cover categories for the years 2004–2006, which are likely descriptors of species natural habitats and showed a reasonable spatial representation in both study areas (i.e. sparse vegetation, bare, rocky and sandy areas). The remaining two habitat variables were constructed from spatial representation of water features (secondary rivers and rock pools) which were digitized from the cartographic maps [92]. Distance to these six habitat categories was computed using the “Euclidian distance” function from ArcGIS. For the North African region, a total of 125 records for *J. jaculus* and 122 records for *J. hirtipes* were included, after reducing spatial clustering by removing records located at lower than ~ 10 km distance from each other using the “occ.desaggragation” function [88]. For the North-West region, a total of 59 records for *J. jaculus* and 97 *J. hirtipes* were retained, using ~ 1 km as distance threshold to remove records and reduce spatial clustering. In both scales, the background area was delimited accordingly to a minimum convex polygon.

## Supplementary information

**Additional file 1:** Microsatellite optimization. Supplementary Tables and Figures.

## Data Availability

DNA sequences and microsatellite genotypes are provided in the supplementary data online with the following link: 10.6084/m9.figshare.9170966.
