# Supplementary material for: Evolutionary history of two cryptic species of northern African jerboas
Source: BMC Evol Biol. 2020 Feb 13;20:26. doi: 10.1186/s12862-020-1592-z (PMC7020373; doi:10.1186/s12862-020-1592-z)
Supplement: Supplementary file 1 — Additional file 1: Microsatellite optimization. Supplementary Tables and Figures. [file 12862_2020_1592_MOESM1_ESM.pdf]

# Supplementary information

## Additional file 1: Microsatellite optimization

The aim was to design and optimize microsatellite markers that would amplify on both lineages of our target species. A total set of 40 loci was initially chosen from the database considering the quality rate (primers classified as "best" were favoured), repeat motif (balanced motifs were preferred), high number of repeats (above 11), small estimated product size (sizes below 200 bp due to the limitations of museum samples under PCR amplification methods (1); although these samples were not used in this study, it is one of the aims for future research) and melting temperatures. The selected loci were arranged in multiplexes for optimization. Multiplexes were designed combining markers with similar melting temperatures (maximum range of 5°C) of forward and reverse primers, and with a minimal interval of around 50 to 70 bp of the product size between loci labelled with the same dye colour (FAM, VIC, NED and PET) in order to avoid overlapping of the loci. AUTODIMER v1.0 (2) was used to perform screening analyses for intramolecular hairpins structures and primer dimer formation within each multiplex to avoid high complementary between primers, thus promoting a higher amplification success. Uniplex PCRs were performed when markers failed to amplify in multiplex reactions in order to estimate the loci individual amplification success, optimal conditions and genotypes profile.

All PCRs were performed in 10µl reaction volumes. Multiplex reactions consisted of 5µl of QIAGEN PCR Master Mix, 3µl of pure water, 1µl of primer mix and 1µl of template DNA. Individual PCRs included 5µl of QIAGEN PCR Master Mix, 2.8µl of pure water, 0.4µl of each primer (forward and reverse), 0.4µl of fluorescent tail and 1µl of template DNA. All PCRs were performed using a touchdown (TD) protocol, which varied on each multiplex and individual reaction conditions (see Annex 6). Each PCR reaction was carried out with an initial denaturation step at 95ºC of 15 min, a denaturation at 95ºC for 30s, a TD method (-0.5ºC) with the respective annealing temperature (Annex 6) of 60s, an extension at 72ºC of 30 s, followed by a final extension of 30 min at 60ºC. The number of cycles for each multiplex/individual reaction is displayed in Annex 6. PCR products were run on an ABI 3130xl Genetic Analyser (AB Applied Biosystems) using 1µl of the amplified product for 10µl of formamide +75-400 (-250) LIZ NEW size standard.

## Tables

**Table S1.** Maximum Likelihood estimates (95% posterior density intervals in parentheses) of demographic parameters obtained with IMa2 between *J. jaculus* and *J. hirtipes* (mean generation time of 1 year) without the candidate genes for fur colouration (*MC1R* and *Agouti*). The values are in millions.

| $N_{e1}$ | $N_{e2}$ | $N_A$ | t | $2Nm_1$ | $2Nm_2$ |
|---|---|---|---|---|---|
| 7.01<br>(5.08-9.93) | 5.66<br>(3.79-8.405) | 4.85<br>(1.67-9.30) | 4.73<br>(0.44-7.65) | 0.14***<br>(0–0.38) | 0.24***<br>(0.04-0.43) |

$N_{e1}$, effective population size of *J. jaculus*; $N_{e2}$, effective population size of *J. hirtipes*; $N_A$, effective population size of the ancestral population; **t**, time since the split between *J. jaculus* and *J. hirtipes*; $2Nm_1$, population migration rate into *J. jaculus*; $2Nm_2$, population migration rate into *J. hirtipes*. Significant values indicated ***(P<0.001).

**Table S2.** P-values and standard error (se) for deviations of the Hardy-Weinberg equilibrium, for excess and deficiency of heterozygotes. The p-value for this analysis was obtained with the Bonferroni correction. Significance levels are indicated: * p-value < 0.002273

| | ***J. jaculus*** | | | | ***J. hirtipes*** | | | |
|---|---|---|---|---|---|---|---|---|
| **Locus** | **Heterozygote deficiency** | | **Heterozygote excess** | | **Heterozygote deficiency** | | **Heterozygote excess** | |
| | **p-value** | **se** | **p-value** | **se** | **p-value** | **se** | **p-value** | **se** |
| **Jac11** | 0.0000* | 0.0000 | 1.00 | 0.0000 | 0.1379 | 0.0238 | 0.8622 | 0.0238 |
| **Jac18** | 0.1873 | 0.0145 | 0.8157 | 0.0142 | 0.0428 | 0.0115 | 0.9572 | 0.0115 |
| **Jac27** | 0.0000* | 0.0000 | 1.00 | 0.0000 | 0.0437 | 0.0181 | 0.9563 | 0.0181 |
| **Jac12** | 0.0018* | 0.0016 | 0.9982 | 0.0016 | 0.0000* | 0.0000 | 1.00 | 0.0000 |
| **Jac37** | 0.1109 | 0.0206 | 0.8901 | 0.0205 | 0.4944 | 0.0310 | 0.5100 | 0.0309 |
| **Jac02** | 0.3059 | 0.0371 | 0.6999 | 0.0375 | 0.5673 | 0.0339 | 0.4328 | 0.0339 |
| **Jac04** | 0.0020* | 0.0007 | 0.9980 | 0.0007 | 0.0166 | 0.0036 | 0.9835 | 0.0036 |
| **Jac07** | 0.6960 | 0.0354 | 0.3315 | 0.0368 | 0.0000* | 0.0000 | 1.00 | 0.0000 |
| **Jac08** | 0.6659 | 0.0290 | 0.3478 | 0.0292 | 0.1495 | 0.0218 | 0.8505 | 0.0218 |
| **Jac23** | 0.0914 | 0.0234 | 0.9095 | 0.0231 | 0.2382 | 0.0243 | 0.7618 | 0.0243 |
| **Jac24** | 0.1139 | 0.0218 | 0.8917 | 0.0211 | 0.0005* | 0.0005 | 0.9995 | 0.0005 |

**Table S3.** Results of Structure Harvester for each number of clusters (K) analysed.

| K | Mean LnP(K) | Stdev LnP(K) | Delta K |
|---|---|---|---|
| **1** | -3617.960000 | 0.733485 | — |
| **2** | -3123.180000 | 0.432435 | 1094.916084 |
| **3** | -3101.880000 | 2.265392 | 12.006753 |
| **4** | -3107.780000 | 5.481514 | 6.826581 |
| **5** | -3151.100000 | 11.777733 | 3.858128 |
| **6** | -3239.860000 | 55.360121 | 1.061053 |
| **7** | -3387.360000 | 82.978690 | 0.046036 |
| **8** | -3531.040000 | 34.125401 | 2.548835 |
| **9** | -3587.740000 | 64.127358 | 1.356987 |
| **10** | -3731.460000 | 100.459012 | — |

**Mean LnP(K):** mean likelihood of each value of K; **Stdev LnP(K):** standard deviation of each likelihood; **Delta K: l**ikelihood of each cluster according to the Evanno method (3)

**Table S4.** List of the complete *Jaculus* samples used in analysis. The two columns labelled as "Cytb (short/long fragment)" and "Nuclear markers" indicate the samples that successfully amplified for at least one of the markers. The respective mitochondrial clade is displayed. *J. orientalis* samples used are also included. The samples geographic distribution is shown in Figure 1; although for some samples the exact coordinates are not available.

ADRA2B (*alpha-2B adrenergic receptor*); GHR (*growth hormone receptor*); IRBP (*interstitial retinoid binding protein*); υWF (*von Willebrand factor*); DBX5 (intron 5 from the *DBX* gene); MC1R (*melanocortin 1 receptor*); *Agouti*; M (microsatellites).

| Sample Code | Museum/Field Collections | Country | Latitude | Longitude | Clade | Cytb (short/long fragment) | Nuclear markers |
|---|---|---|---|---|---|---|---|
| **ZBSC 0013** | Mauritania 2010 | Morocco | 28,829 | -10,266 | 2 | | |
| **ZBSC 0019** | Mauritania 2010 | Mauritania | 20,997 | -16,283 | 1 | Short/Long | ADRA2B, GHR, IRBP, VWF, DBX5, MC1R, Agouti, M |
| **ZBSC 0020** | Mauritania 2010 | Mauritania | 20,929 | -16,221 | 2 | Short/Long | ADRA2B, GHR, IRBP, VWF, DBX5, MC1R, Agouti, M |
| **ZBSC 0021** | Mauritania 2010 | Mauritania | 20,602 | -16,012 | 1 | Short/Long | ADRA2B, GHR, IRBP, VWF, DBX5, MC1R, Agouti, M |
| **ZBSC 0027** | Mauritania 2010 | Mauritania | 16,566 | -14,198 | 2 | | |
| **ZBSC 0028** | Mauritania 2010 | Mauritania | 16,435 | -14,037 | 2 | | |
| **ZBSC 0064** | Mauritania 2010 | Mauritania | 18,901 | -15,416 | 1 | Short/Long | ADRA2B, GHR, IRBP, VWF, DBX5, MC1R, Agouti, M |
| **ZBSC 0070** | Mauritania 2010 | Mauritania | 20,724 | -16,057 | 2 | Short/Long | ADRA2B, DBX5, M |
| **ZBSC 0072** | Mauritania 2010 | Mauritania | 20,379 | -15,991 | 1 | Short/Long | ADRA2B, GHR, IRBP, VWF, DBX5, M |

| | | | | | | | |
|---|---|---|---|---|---|---|---|
| **ZBSC 0079** | Mauritania 2010 | Mauritania | 20,613 | -16,013 | 1 | Short/Long | ADRA2B, GHR, IRBP, VWF, DBX5, MC1R, Agouti, M |
| **ZBSC 0081** | Mauritania 2010 | Western Sahara | 22,639 | -16,337 | 2 | Short/Long | ADRA2B, GHR, IRBP, VWF, DBX5, MC1R, Agouti, M |
| **ZBSC 0082** | Mauritania 2010 | Western Sahara | 24,297 | -15,333 | 2 | Short/Long | ADRA2B, GHR, IRBP, VWF, DBX5, MC1R, Agouti, M |
| **ZBSC 0083** | Mauritania 2010 | Western Sahara | 24,630 | -14,945 | 2 | Short/Long | ADRA2B, GHR, IRBP, VWF, DBX5, MC1R, Agouti, M |
| **ZBSC 0084** | Mauritania 2010 | Western Sahara | 25,322 | -14,795 | 2 | Short/Long | ADRA2B, GHR, IRBP, VWF, DBX5, MC1R, Agouti, M |
| **ZBSC 0193** | Mauritania 2011 | Western Sahara | 25,267 | -14,821 | 2 | Short/Long | ADRA2B, GHR, IRBP, VWF, DBX5, MC1R, Agouti, M |
| **ZBSC 0196** | Mauritania 2011 | Western Sahara | 24,006 | -15,611 | 2 | Short/Long | ADRA2B, DBX5, M |
| **ZBSC 0197** | Mauritania 2011 | Western Sahara | 22,829 | -16,250 | 2 | Short/Long | ADRA2B, GHR, IRBP, VWF, DBX5, Agouti, M |
| **ZBSC 0198** | Mauritania 2011 | Western Sahara | 22,557 | -16,370 | 2 | Short/Long | ADRA2B, GHR, IRBP, VWF, DBX5, Agouti, M |
| **ZBSC 0218** | Mauritania 2011 | Mauritania | 21,438 | -12,980 | 1 | Short/Long | ADRA2B, GHR, IRBP, VWF, DBX5, MC1R, Agouti, M |

| | | | | | | | |
|---|---|---|---|---|---|---|---|
| **ZBSC 0219** | Mauritania 2011 | Mauritania | 21,352 | -13,039 | 2 | Short/Long | ADRA2B, GHR, IRBP, VWF, DBX5, MC1R, Agouti, M |
| **ZBSC 0224** | Mauritania 2011 | Mauritania | 20,557 | -12,572 | 2 | Short/Long | ADRA2B, GHR, IRBP, VWF, DBX5, MC1R, Agouti, M |
| **ZBSC 0226** | Mauritania 2011 | Mauritania | 20,508 | -12,831 | 2 | Short/Long | ADRA2B, GHR, IRBP, VWF, DBX5, MC1R, Agouti, M |
| **ZBSC 0240** | Mauritania 2011 | Mauritania | 20,254 | -13,296 | 2 | Short/Long | ADRA2B, GHR, IRBP, VWF, DBX5, M |
| **ZBSC 0241** | Mauritania 2011 | Mauritania | 20,253 | -13,311 | 1 | Short/Long | ADRA2B, GHR, IRBP, VWF, DBX5, MC1R, Agouti, M |
| **ZBSC 0242** | Mauritania 2011 | Mauritania | 20,016 | -13,887 | 2 | Short/Long | ADRA2B, GHR, IRBP, DBX5, MC1R, Agouti, M |
| **ZBSC 0243** | Mauritania 2011 | Mauritania | 19,651 | -14,504 | 1 | Short/Long | ADRA2B, GHR, IRBP, VWF, DBX5, MC1R, Agouti, M |
| **ZBSC 0244** | Mauritania 2011 | Mauritania | 19,651 | -14,504 | 1 | Short/Long | ADRA2B, GHR, IRBP, VWF, DBX5, MC1R, Agouti, M |
| **ZBSC 0245** | Mauritania 2011 | Mauritania | 19,651 | -14,504 | 2 | Short/Long | ADRA2B, GHR, IRBP, VWF, DBX5, MC1R, Agouti, M |
| **ZBSC 0246** | Mauritania 2011 | Mauritania | 19,651 | -14,504 | 1 | Short | GHR |
| **ZBSC 0256** | Mauritania 2011 | Mauritania | 17,591 | -12,848 | 1 | Short/Long | ADRA2B, DBX5, M |

| | | | | | | | |
|---|---|---|---|---|---|---|---|
| **ZBSC 0265** | Mauritania 2011 | Mauritania | 18,094 | -12,132 | 1 | Short/Long | ADRA2B, GHR, IRBP, VWF, DBX5, MC1R, Agouti, M |
| **ZBSC 0267** | Mauritania 2011 | Western Sahara | 22,136 | -16,570 | 2 | Short/Long | GHR, DBX5, MC1R, Agouti, |
| **ZBSC 0290** | Mauritania 2012 | Western Sahara | 27,149 | -10,847 | | | |
| **ZBSC 0291** | Mauritania 2012 | Western Sahara | 27,055 | -11,410 | 2 | Short/Long | ADRA2B, GHR, IRBP, VWF, DBX5, MC1R, Agouti, M |
| **ZBSC 0292** | Mauritania 2012 | Western Sahara | 26,959 | -11,657 | 2 | Short/Long | ADRA2B, GHR, IRBP, VWF, DBX5, MC1R, Agouti, M |
| **ZBSC 0293** | Mauritania 2012 | Western Sahara | 26,933 | -11,700 | 2 | Short/Long | ADRA2B, GHR, IRBP, VWF, DBX5, MC1R, Agouti, M |
| **ZBSC 0294** | Mauritania 2012 | Western Sahara | 26,811 | -11,746 | 2 | Short/Long | ADRA2B, GHR, IRBP, VWF, DBX5, MC1R, Agouti, M |
| **ZBSC 0295** | Mauritania 2012 | Western Sahara | 25,246 | -12,488 | 2 | Short/Long | ADRA2B, GHR, IRBP, VWF, DBX5, MC1R, Agouti, M |
| **ZBSC 0296** | Mauritania 2012 | Western Sahara | 27,167 | -10,964 | 2 | Short/Long | ADRA2B, GHR, IRBP, VWF, DBX5, Agouti, M |
| **ZBSC 0303** | Mauritania 2012 | Mauritania | 21,021 | -16,304 | 1 | Short | GHR, IRBP, VWF, DBX5, M |
| **ZBSC 0306** | Mauritania 2012 | Mauritania | 16,633 | -15,196 | 2 | Short/Long | ADRA2B, GHR, IRBP, VWF, DBX5, MC1R, Agouti, M |

| | | | | | | | |
|---|---|---|---|---|---|---|---|
| **ZBSC 0382** | Mauritania: October/December 2012 | Morocco | 32,474 | -4,494 | | | |
| **ZBSC 0383** | Mauritania: October/December 2012 | Morocco | 28,961 | -10,508 | 2 | Short/Long | ADRA2B, GHR, IRBP, VWF, DBX5, MC1R, Agouti, M |
| **ZBSC 0384** | Mauritania: October/December 2012 | Morocco | 28,394 | -11,026 | 2 | Short/Long | ADRA2B, GHR, IRBP, VWF, DBX5, MC1R, Agouti, M |
| **ZBSC 0385** | Morocco 2012 | Morocco | 28,330 | -10,913 | 2 | Short/Long | ADRA2B, GHR, IRBP, VWF, DBX5, Agouti, M |
| **ZBSC 0388** | Morocco 2012 | Morocco | 28,823 | -10,371 | 2 | Short/Long | ADRA2B, GHR, IRBP, VWF, DBX5, MC1R, Agouti, M |
| **ZBSC 0403** | Mauritania 2014 | Western Sahara | 25,936 | -14,514 | 2 | Short/Long | ADRA2B, GHR, IRBP, VWF, DBX5, MC1R, Agouti, M |
| **ZBSC 0404** | Mauritania 2014 | Western Sahara | 25,654 | -14,660 | 2 | Short/Long | ADRA2B, GHR, IRBP, VWF, DBX5, MC1R, Agouti, M |
| **ZBSC 0408** | Mauritania 2014 | Western Sahara | 23,846 | -15,863 | 2 | Short/Long | ADRA2B, GHR, IRBP, VWF, DBX5, MC1R, Agouti, M |
| **ZBSC 0413** | Mauritania 2014 | Western Sahara | 23,595 | -15,715 | 2 | Short/Long | ADRA2B, GHR, IRBP, VWF, DBX5, MC1R, Agouti, M |
| **ZBSC 0414** | Mauritania 2014 | Western Sahara | 23,115 | -14,964 | 2 | Short/Long | ADRA2B, GHR, IRBP, VWF, DBX5, MC1R, Agouti, M |

| | | | | | | | |
|---|---|---|---|---|---|---|---|
| **ZBSC 0417** | Mauritania 2014 | Mauritania | 20,844 | -16,149 | 1 | Short/Long | ADRA2B, GHR, IRBP, VWF, DBX5, MC1R, Agouti, M |
| **ZBSC 0420** | Mauritania 2014 | Mauritania | 20,093 | -15,927 | 1 | Short/Long | ADRA2B, GHR, IRBP, VWF, DBX5, MC1R, Agouti, M |
| **ZBSC 0423** | Mauritania 2014 | Mauritania | 16,220 | -13,260 | 2 | Short/Long | ADRA2B, GHR, IRBP, VWF, DBX5, MC1R, Agouti, M |
| **ZBSC 0424** | Mauritania 2014 | Mauritania | 15,565 | -12,327 | 2 | Short/Long | ADRA2B, GHR, IRBP, VWF, DBX5, MC1R, Agouti, M |
| **ZBSC 0509** | Mauritania 2014 | Mauritania | 18,383 | -9,313 | 1 | Short/Long | ADRA2B, GHR, IRBP, VWF, DBX5, MC1R, Agouti, M |
| **ZBSC 0526** | Mauritania 2014 | Mauritania | 18,559 | -11,248 | 1 | Short/Long | ADRA2B, GHR, IRBP, VWF, DBX5, MC1R, Agouti, M |
| **ZBSC 0542** | Mauritania 2014 | Mauritania | 18,357 | -11,816 | 1 | Short/Long | ADRA2B, GHR, IRBP, VWF, DBX5, MC1R, Agouti, M |
| **ZBSC 0543** | Mauritania 2014 | Mauritania | 18,357 | -11,816 | 1 | Short/Long | ADRA2B, GHR, IRBP, VWF, DBX5, MC1R, Agouti, M |
| **ZBSC 0558** | Mauritania 2014 | Mauritania | 18,109 | -11,916 | 1 | Short/Long | ADRA2B, GHR, IRBP, VWF, DBX5, MC1R, Agouti, M |
| **ZBSC 0559** | Mauritania 2014 | Mauritania | 18,000 | -11,884 | 1 | Short/Long | ADRA2B, GHR, IRBP, VWF, DBX5, MC1R, Agouti, M |

| | | | | | | | |
|---|---|---|---|---|---|---|---|
| **ZBSC 0591** | Mauritania 2014 | Morocco | 32,255 | -2,215 | 2 | Short/Long | ADRA2B, GHR, IRBP, VWF, DBX5, M |
| **ZBSC 0599** | Mauritania 2014 | Morocco | 32,156 | -1,327 | 2 | Short/Long | ADRA2B, GHR, IRBP, VWF, DBX5, MC1R, Agouti, M |
| **ZBSC 0614** | Mauritania 2014 | Morocco | 28,393 | -11,028 | 2 | Short/Long | ADRA2B, GHR, IRBP, VWF, DBX5, MC1R, Agouti, M |
| **ZBSC 0626** | Mauritania 2014 | Morocco | 28,179 | -11,857 | 2 | Short/Long | ADRA2B, GHR, IRBP, VWF, DBX5, MC1R, Agouti, M |
| **ZBSC 0629** | Mauritania 2014 | Morocco | 28,559 | -10,918 | 2 | Short/Long | ADRA2B, GHR, IRBP, VWF, DBX5, MC1R, Agouti, M |
| **ZBSC 0647** | Morocco winter 2015 | Morocco | 27,924 | -11,444 | 2 | Short/Long | ADRA2B, GHR, IRBP, VWF, DBX5, MC1R, Agouti, M |
| **ZBSC 0650** | Morocco winter 2015 | Morocco | 27,907 | -11,600 | 2 | Short/Long | ADRA2B, GHR, IRBP, VWF, DBX5, MC1R, Agouti, M |
| **ZBSC 0651** | Morocco winter 2015 | Morocco | 27,895 | -11,610 | 2 | Short/Long | ADRA2B, GHR, IRBP, VWF, DBX5, MC1R, Agouti, M |
| **ZBSC 0652** | Morocco winter 2015 | Morocco | 27,901 | -11,605 | 2 | Short/Long | ADRA2B, GHR, IRBP, VWF, DBX5, MC1R, Agouti, M |
| **ZBSC 0653** | Morocco winter 2015 | Morocco | 27,875 | -11,588 | 2 | Short/Long | ADRA2B, GHR, IRBP, VWF, DBX5, MC1R, Agouti, M |

| | | | | | | | |
|---|---|---|---|---|---|---|---|
| **ZBSC 0654** | Morocco winter 2015 | Morocco | 27,907 | -11,600 | 2 | Short/Long | ADRA2B, GHR, IRBP, VWF, DBX5, MC1R, Agouti, M |
| **ZBSC 0665** | Morocco winter 2015 | Morocco | 27,224 | -12,883 | 2 | Short/Long | ADRA2B, GHR, IRBP, VWF, DBX5, MC1R, Agouti, M |
| **ZBSC 0675** | Morocco winter 2015 | Morocco | 26,579 | -12,769 | 2 | Short/Long | ADRA2B, GHR, IRBP, VWF, DBX5, MC1R, Agouti, M |
| **ZBSC 0678** | Morocco winter 2015 | Morocco | 27,046 | -11,537 | 2 | Short/Long | ADRA2B, GHR, IRBP, VWF, DBX5, MC1R, Agouti, M |
| **ZBSC 0679** | Morocco winter 2015 | Morocco | 27,054 | -11,432 | 2 | Short/Long | ADRA2B, GHR, IRBP, VWF, DBX5, Agouti, M |
| **ZBSC 0681** | Morocco winter 2015 | Morocco | 27,076 | -11,756 | 2 | Short/Long | ADRA2B, GHR, IRBP, VWF, DBX5, MC1R, Agouti, M |
| **ZBSC 0683** | Morocco winter 2015 | Morocco | 27,386 | -11,694 | 2 | Short/Long | MC1R, Agouti, |
| **ZBSC 0684** | Morocco winter 2015 | Morocco | 27,453 | -11,681 | 2 | Short/Long | ADRA2B, GHR, IRBP, VWF, DBX5, MC1R, Agouti, M |
| **ZBSC 0685** | Morocco winter 2015 | Morocco | 27,453 | -11,681 | 2 | Short/Long | ADRA2B, GHR, IRBP, VWF, DBX5, MC1R, Agouti, M |
| **ZBSC 0686** | Morocco winter 2015 | Morocco | 27,575 | -11,634 | 2 | Short/Long | ADRA2B, GHR, IRBP, VWF, DBX5, MC1R, Agouti, M |

| | | | | | | | |
|---|---|---|---|---|---|---|---|
| **ZBSC 0688** | Morocco winter 2015 | Morocco | 27,938 | -11,577 | 2 | Short/Long | ADRA2B, GHR, IRBP, VWF, DBX5, MC1R, Agouti, M |
| **ZBSC 0690** | Morocco winter 2015 | Morocco | 27,902 | -11,605 | 2 | Short/Long | ADRA2B, GHR, IRBP, VWF, DBX5, MC1R, Agouti, M |
| **ZBSC 0691** | Morocco winter 2015 | Morocco | 27,918 | -11,553 | 2 | Short/Long | ADRA2B, GHR, IRBP, VWF, DBX5, MC1R, Agouti, M |
| **ZBSC 0692** | Morocco winter 2015 | Morocco | 27,918 | -11,554 | 2 | Short/Long | ADRA2B, GHR, IRBP, VWF, DBX5, MC1R, Agouti, M |
| **ZBSC 0693** | Morocco winter 2015 | Morocco | 27,914 | -11,545 | 2 | Short/Long | ADRA2B, GHR, IRBP, VWF, DBX5, MC1R, Agouti, M |
| **ZBSC 0694** | Morocco winter 2015 | Morocco | 27,902 | -11,604 | 2 | Short/Long | ADRA2B, GHR, IRBP, VWF, DBX5, MC1R, Agouti, M |
| **ZBSC 0695** | Morocco winter 2015 | Morocco | 27,927 | -11,571 | 2 | Short/Long | ADRA2B, GHR, IRBP, VWF, DBX5, MC1R, Agouti, M |
| **ZBSC 0696** | Morocco winter 2015 | Morocco | 27,907 | -11,601 | 2 | Short/Long | ADRA2B, GHR, IRBP, VWF, DBX5, MC1R, Agouti, M |
| **ZBSC 0697** | Morocco winter 2015 | Morocco | 27,938 | -11,577 | 2 | Short/Long | ADRA2B, GHR, IRBP, VWF, DBX5, MC1R, Agouti, M |
| **ZBSC 0699** | Morocco winter 2015 | Morocco | 27,926 | -11,444 | 2 | Short/Long | ADRA2B, GHR, IRBP, VWF, DBX5, MC1R, Agouti, M |

| | | | | | | | |
|---|---|---|---|---|---|---|---|
| **ZBSC 0703** | Morocco winter 2015 | Morocco | 28,110 | -11,301 | 2 | Short/Long | ADRA2B, GHR, IRBP, VWF, DBX5, MC1R, Agouti, M |
| **D100** | National Geographic:Sept-Decem 2004 | Mauritania | 17,938 | -12,267 | 2 | Short/Long | ADRA2B, GHR, IRBP, VWF, DBX5, MC1R, Agouti, M |
| **D1283** | National Geographic:Sept-Decem 2004 | Western Sahara | 22,446 | -16,448 | 2 | Short/Long | ADRA2B, GHR, IRBP, VWF, DBX5, MC1R, Agouti, M |
| **D144** | National Geographic:Sept-Decem 2004 | Western Sahara | 24,847 | -14,844 | 2 | Short/Long | ADRA2B, GHR, IRBP, DBX5, Agouti, M |
| **D145** | National Geographic:Sept-Decem 2004 | Western Sahara | 25,245 | -14,821 | 2 | Short/Long | ADRA2B, GHR, IRBP, VWF, DBX5, M |
| **D535** | National Geographic:Sept-Decem 2004 | Western Sahara | 21,937 | -16,875 | 2 | Short/Long | ADRA2B, VWF |
| **D549** | National Geographic:Sept-Decem 2004 | Western Sahara | 24,788 | -14,865 | 2 | Short/Long | ADRA2B, GHR, IRBP, VWF, DBX5, M |
| **D576** | National Geographic:Sept-Decem 2004 | Morocco | 29,389 | -8,129 | 2 | Short/Long | ADRA2B, GHR, IRBP, VWF, DBX5, M |
| **D577** | National Geographic:Sept-Decem 2004 | Morocco | 30,038 | -6,894 | 2 | Short/Long | ADRA2B, GHR, IRBP, VWF, DBX5, MC1R, Agouti, M |
| **D578** | National Geographic:Sept-Decem 2004 | Morocco | 30,038 | -6,894 | 2 | Short/Long | ADRA2B, GHR, IRBP, VWF, DBX5, M |

| | | | | | | | |
|---|---|---|---|---|---|---|---|
| **D796** | National Geographic:Sept-Decem 2004 | Western Sahara | 24,686 | -14,862 | 2 | Short/Long | ADRA2B, GHR, VWF, DBX5 |
| **D800** | National Geographic:Sept-Decem 2004 | Western Sahara | 25,306 | -14,803 | 2 | Short/Long | ADRA2B, GHR, IRBP, VWF, M |
| **D684** | National Geographic:Sept-Decem 2004 | Morocco | 31,075 | -4,011 | 2 | Short/Long | ADRA2B, GHR, IRBP, VWF, MC1R, Agouti, DBX5 |
| **D53** | National Geographic:Sept-Decem 2004 | Mauritania | 17,195 | -7,141 | 1 | Short/Long | ADRA2B, GHR, IRBP, VWF, DBX5, M |
| **D52** | National Geographic:Sept-Decem 2004 | Mauritania | 17,225 | -7,069 | 1 | Short/Long | ADRA2B, GHR, IRBP, VWF, DBX5, MC1R, M |
| **D117** | National Geographic:Sept-Decem 2004 | Mauritania | 17,393 | -13,453 | 1 | Short/Long | GHR, DBX5 |
| **D493** | National Geographic:Sept-Decem 2004 | Mauritania | 17,408 | -16,062 | 1 | Short/Long | ADRA2B, GHR, IRBP, VWF, DBX5, M |
| **D113** | National Geographic:Sept-Decem 2004 | Mauritania | 17,693 | -12,571 | 1 | Short/Long | ADRA2B, GHR, IRBP, VWF, DBX5, MC1R, Agouti, M |
| **D3055** | National Geographic:Sept-Decem 2004 | Mauritania | 17,895 | -11,716 | 1 | Short/Long | ADRA2B, GHR, IRBP, VWF, DBX5, MC1R, Agouti, M |
| **D22** | National Geographic:Sept-Decem 2004 | Mauritania | 17,899 | -12,334 | 1 | Short/Long | ADRA2B, GHR, VWF, DBX5, M |
| **D101** | National Geographic:Sept-Decem 2004 | Mauritania | 17,938 | -12,267 | 1 | Short/Long | ADRA2B, GHR, IRBP, VWF, DBX5, MC1R, Agouti, M |

| | | | | | | | |
|---|---|---|---|---|---|---|---|
| **D3107** | National Geographic:Sept-Decem 2004 | Mauritania | 18,021 | -12,050 | 1 | Short/Long | ADRA2B, GHR, IRBP, VWF, DBX5, Agouti, M |
| **D506** | National Geographic:Sept-Decem 2004 | Mauritania | 19,439 | -14,754 | 1 | Short/Long | ADRA2B, GHR, IRBP, VWF, Agouti |
| **D511** | National Geographic:Sept-Decem 2004 | Mauritania | 19,641 | -14,522 | 1 | Short/Long | ADRA2B, GHR, IRBP, VWF, DBX5, M |
| **D1003** | National Geographic:Sept-Decem 2004 | Mauritania | 20,378 | -15,991 | 1 | Short/Long | ADRA2B, GHR, IRBP, VWF, DBX5, MC1R, Agouti, M |
| **D1630** | National Geographic:Sept-Decem 2004 | Mauritania | 21,355 | -13,025 | 1 | | |
| **D945** | National Geographic:Sept-Decem 2004 | Morocco | 28,633 | -10,753 | 1 | | |
| **D320** | National Geographic:Sept-Decem 2004 | Tunisia | 33,014 | 10,952 | 1 | Short/Long | ADRA2B, GHR, IRBP, VWF, DBX5, MC1R, Agouti, M |
| **D536** | National Geographic:Sept-Decem 2004 | Western Sahara | 21,969 | -16,874 | 1 | Short/Long | ADRA2B, GHR, IRBP, VWF, DBX5, MC1R, Agouti, M |
| **D541** | National Geographic:Sept-Decem 2004 | Western Sahara | 22,367 | -16,462 | 1 | Short/Long | ADRA2B, GHR, IRBP, VWF, DBX5, MC1R, Agouti, M |
| **D316** | National Geographic:Sept-Decem 2004 | Tunisia | 33,498 | 9,383 | 2 | | ADRA2B, GHR, VWF |
| **8067** | Collected by Luis Garcia-Cardenete | Morocco | 28,991 | -10,315 | 2 | Short/Long | ADRA2B, GHR, IRBP, VWF, DBX5, MC1R, Agouti, M |

| | | | | | | | |
|---|---|---|---|---|---|---|---|
| **8069** | Collected by Luis Garcia-Cardenete | Morocco | 28,598 | -10,859 | 2 | Short/Long | ADRA2B, GHR, IRBP, VWF, DBX5, MC1R, Agouti, M |
| **8070** | Collected by Luis Garcia-Cardenete | Morocco | 28,298 | -11,189 | 2 | Short/Long | ADRA2B, GHR, IRBP, VWF, DBX5, Agouti, M |
| **8072** | Collected by Luis Garcia-Cardenete | Morocco | 27,723 | -11,565 | 2 | Short/Long | ADRA2B, GHR, IRBP, VWF, DBX5, M |
| **8076** | Collected by Luis Garcia-Cardenete | Western Sahara | 26,689 | -11,854 | 2 | Short/Long | ADRA2B, GHR, IRBP, VWF, DBX5, MC1R, Agouti, M |
| **8345** | Collected by Luis Garcia-Cardenete | Morocco | 32,416 | -2,087 | 2 | Short/Long | ADRA2B, GHR, IRBP, VWF, DBX5, MC1R, Agouti, M |
| **8350** | Collected by Luis Garcia-Cardenete | Morocco | 32,141 | -2,791 | 2 | Short/Long | ADRA2B, GHR, IRBP, VWF, DBX5, MC1R, Agouti, M |
| **8367** | Collected by Luis Garcia-Cardenete | Morocco | 29,825 | -7,524 | 2 | Short/Long | ADRA2B, GHR, IRBP, VWF, DBX5, MC1R, Agouti, M |
| **8374** | Collected by Luis Garcia-Cardenete | Morocco | 28,905 | -10,170 | 2 | Short/Long | ADRA2B, GHR, IRBP, VWF, DBX5, M |
| **8545** | Observed by F. Martínez-Freiría | Morocco | 31,144 | -7,404 | | Short/Long | ADRA2B, GHR, IRBP, VWF, DBX5, M |
| **9072** | Collected by LG Cardenete | Morocco | 28,069 | -11,361 | 2 | Short/Long | ADRA2B, GHR, IRBP, VWF, DBX5, MC1R, Agouti, M |

| | | | | | | | |
|---|---|---|---|---|---|---|---|
| **9083** | Collected by LG Cardenete | Morocco | 28,361 | -10,859 | 2 | Short/Long | ADRA2B, GHR, IRBP, VWF, DBX5, MC1R, Agouti, M |
| **9086** | Collected by LG Cardenete | Morocco | 29,010 | -10,207 | 2 | Short/Long | ADRA2B, GHR, IRBP, VWF, DBX5, MC1R, Agouti, M |
| **9087** | Collected by LG Cardenete | Morocco | 28,655 | -10,486 | 2 | Short/Long | ADRA2B, GHR, IRBP, VWF, DBX5, MC1R, M |
| **10355** | F Álvares, R Godinho, M Nakamura, J Layna | Senegal | 16,355 | -16,224 | 2 | Short/Long | ADRA2B, GHR, IRBP, VWF, DBX5, MC1R, Agouti, M |
| **6064** | Mauritania 2011 | Mauritania | 18,236 | -11,519 | 1 | Short/Long | ADRA2B, GHR, IRBP, VWF, DBX5, MC1R, Agouti, M |
| **6269** | Mauritania 2011 | Mauritania | 18,480 | -16,022 | 1 | Short/Long | ADRA2B, GHR, IRBP, VWF, DBX5, MC1R, Agouti, M |
| **6367** | Mauritania 2011 | Mauritania | 19,960 | -16,084 | 1 | Short/Long | ADRA2B, GHR, IRBP, VWF, DBX5, M MC1R, Agouti, |
| **6267** | Mauritania 2011 | Mauritania | 18,480 | -16,022 | 1 | | |
| **6268** | Mauritania 2011 | Mauritania | 18,480 | -16,022 | 1 | Short | ADRA2B, GHR, IRBP, VWF, DBX5, MC1R, Agouti, M |
| **6481** | Mauritania 2011 | Western Sahara | 22,276 | -16,494 | 2 | | ADRA2B, GHR, IRBP, VWF, DBX5, MC1R, Agouti, M |

| | | | | | | | |
|---|---|---|---|---|---|---|---|
| **6482** | Mauritania 2011 | Western Sahara | 22,348 | -16,469 | 2 | Short/Long | ADRA2B, GHR, IRBP, VWF, DBX5, MC1R, Agouti, M |
| **1221** | Mauritania 2011 | Morocco | 31,075 | -4,011 | | | |
| **11043** | Mauritania: November 2014 | Mauritania | 16,068 | -11,509 | 1 | Short/Long | ADRA2B, GHR, IRBP, VWF, DBX5, MC1R, Agouti, M |
| **11379** | Mauritania: November 2014 | Mauritania | 18,197 | -15,047 | 1 | Short/Long | ADRA2B, GHR, IRBP, VWF, DBX5, MC1R, Agouti, M |
| **11382** | Mauritania: November 2014 | Mauritania | 18,261 | -14,981 | 1 | Short/Long | ADRA2B, GHR, IRBP, VWF, DBX5, MC1R, Agouti, M |
| **11384** | Mauritania: November 2014 | Mauritania | 18,490 | -14,644 | 1 | Short/Long | ADRA2B, GHR, IRBP, VWF, DBX5, MC1R, Agouti, M |
| **11408** | Mauritania: November 2014 | Mauritania | 18,429 | -14,801 | 1 | Short/Long | ADRA2B, GHR, IRBP, VWF, DBX5, MC1R, Agouti, M |
| **11411** | Mauritania: November 2014 | Western Sahara | 22,061 | -16,736 | 2 | Short/Long | ADRA2B, GHR, IRBP, VWF, DBX5, MC1R, Agouti, M |
| **37709** | Royal Museum of Central Africa Tervuren | Egypt | 22,004 | 25,145 | 1 | Short | |
| **37711** | Royal Museum of Central Africa Tervuren | Egypt | 22,004 | 25,145 | | | |
| **37720** | Royal Museum of Central Africa Tervuren | Egypt | 22,004 | 25,145 | 1 | Short | |
| **37728** | Royal Museum of Central Africa Tervuren | Egypt | 22,004 | 25,145 | 1 | Short/Long | |
| **37731** | Royal Museum of Central Africa Tervuren | Egypt | 22,004 | 25,145 | 1 | Short | |
| **37750** | Royal Museum of Central Africa Tervuren | Egypt | 22,030 | 25,141 | 1 | Short/Long | |

| | | | | | | | |
|---|---|---|---|---|---|---|---|
| **37761** | Royal Museum of Central Africa Tervuren | Egypt | 22,037 | 25,097 | 1 | Short | |
| **37762** | Royal Museum of Central Africa Tervuren | Egypt | 22,037 | 25,097 | 1 | Short | |
| **37763** | Royal Museum of Central Africa Tervuren | Egypt | 22,037 | 25,097 | 1 | Short | |
| **37764** | Royal Museum of Central Africa Tervuren | Egypt | 22,004 | 25,145 | 1 | Short | |
| **5046** | Royal Museum of Central Africa Tervuren | Sudan | 15,600 | 32,800 | 1 | Short | |
| **5047** | Royal Museum of Central Africa Tervuren | Sudan | 15,600 | 32,800 | 1 | Short | |
| **13536** | Natural History Museum (Naturhistorisches Museum) – Vienna | Israel | | | 1 | Short/Long | |
| **16984** | Natural History Museum, Brussels, Belgium | Egypt | 22,010 | 25,140 | 1 | Short | |
| **16989** | Natural History Museum, Brussels, Belgium | Egypt | 22,010 | 25,140 | 1 | Short/Long | |
| **16992** | Natural History Museum, Brussels, Belgium | Egypt | 22,037 | 25,096 | 1 | Short | |
| **16993** | Natural History Museum, Brussels, Belgium | Egypt | 22,037 | 25,096 | 1 | Short | |
| **467B** | Natural History Museum, Brussels, Belgium | Saudi-Arabia | | | 1 | Short | |
| **72.64.1.** | Hungarian Museum of natural History | Tunisia | 36,480 | 10,670 | | | |
| **72.64.2.** | Hungarian Museum of natural History | Tunisia | 36,480 | 10,670 | | | |
| **72.64.3.** | Hungarian Museum of natural History | Tunisia | 36,480 | 10,670 | | | |
| **72.64.4.** | Hungarian Museum of natural History | Tunisia | 36,480 | 10,670 | | | |
| **72.64.5.** | Hungarian Museum of natural History | Tunisia | 36,480 | 10,670 | | | |
| **81.54.1** | HNHM, Hungary | Iraq | | | | | |
| **86.28.1.** | Hungarian Museum of natural History | Algeria | 32,833 | 3,767 | | | |
| **88.119.1.** | Hungarian Museum of natural History | Tunisia | 36,480 | 10,670 | | | |
| **2000.75.7** | HNHM, Hungary | Iraq | | | | | |

| | | | | | | | |
|---|---|---|---|---|---|---|---|
| **3302** | MZUF, Florence | Somalia | 4,720 | 46,597 | | | |
| **6262** | MZUF, Florence | Somalia | 8,237 | 48,265 | | | |
| **3299** | MZUF, Florence | Somalia | 4,720 | 46,597 | | | |
| **6261** | MZUF, Florence | Somalia | 4,720 | 46,597 | | | |
| **6296** | MZUF, Florence | Somalia | 9,975 | 50,540 | | | |
| **10010** | MZUF, Florence | Somalia | 4,083 | 46,550 | | | |
| **10011** | MZUF, Florence | Somalia | 4,083 | 46,550 | | | |
| **2484** | MZUF, Florence | Somalia | 4,720 | 46,597 | | | |
| **6260** | MZUF, Florence | Somalia | 4,720 | 46,597 | | | |
| **M/9798/90** | Polish Academy of Science, Museum of Institute of Systematics and Evolution of Mammals | Algeria | 28,250 | 0,200 | 1 | Short | |
| **M/9799/90** | Polish Academy of Science, Museum of Institute of Systematics and Evolution of Mammals | Algeria | 30,717 | 3,133 | 1 | Short | |
| **M/9796/90** | Polish Academy of Science, Museum of Institute of Systematics and Evolution of Mammals | Algeria | 33,100 | 1,267 | | | |
| **M/9795/90** | Polish Academy of Science, Museum of Institute of Systematics and Evolution of Mammals | Algeria | 32,166 | 5,189 | | | |
| **M/9801/90** | Polish Academy of Science, Museum of Institute of Systematics and Evolution of Mammals | Algeria | 35,137 | 3,016 | 1 | Short | |
| **M/9800/90** | Polish Academy of Science, Museum of Institute of Systematics and Evolution of Mammals | Algeria | 23,700 | 5,133 | | | |
| **M/9797/90** | Polish Academy of Science, Museum of Institute of Systematics and Evolution of Mammals | Algeria | 28,250 | -0,200 | 1 | Short | |
| **M/9794/90** | Polish Academy of Science, Museum of Institute of Systematics and Evolution of Mammals | Algeria | 35,094 | 3,017 | | | |

| | | | | | | | | |
|---|---|---|---|---|---|---|---|---|
| **M/9790/90** | Polish Academy of Science, Museum of Institute of Systematics and Evolution of Mammals | Algeria | | | | | | |
| **M/9792/90** | Polish Academy of Science, Museum of Institute of Systematics and Evolution of Mammals | Algeria | | | | | | |
| **5606** | MZUF Florence | Lybia | | | | | | |
| **22020** | Belgium Royal Museum of Natural History Brussels | Tunisia | | | 1 | Short | | |
| **22021** | Belgium Royal Museum of Natural History Brussels | Tunisia | 34,483 | 9,583 | 1 | Short | | |
| **30263** | Natural History Museum (Naturhistorisches Museum) – Vienna | Libya | | | 1 | Short | | |
| **30264** | Natural History Museum (Naturhistorisches Museum) – Vienna | Libya | | | | | | |
| **M-0274** | Belgium Royal Museum of Cantral Africs Tervuren | Tunisia | | | | | | |
| **M-0275** | Belgium Royal Museum of Cantral Africs Tervuren | Tunesia | | | 1 | Short | | |
| **M-0277** | Belgium Royal Museum of Cantral Africs Tervuren | Tunesia | | | | | | |
| **M-0278** | Belgium Royal Museum of Cantral Africs Tervuren | Tunesia | | | | | | |
| **M-0279** | Belgium Royal Museum of Cantral Africs Tervuren | Tunesia | | | | | | |
| **M-0280** | Belgium Royal Museum of Cantral Africs Tervuren | Tunesia | | | | | | |
| **M-0281** | Belgium Royal Museum of Cantral Africs Tervuren | Tunesia | | | 1 | Short | | |
| **M-0282** | Belgium Royal Museum of Cantral Africs Tervuren | Libya | | | 1 | Short | | |
| **M-0283** | Belgium Royal Museum of Cantral Africs Tervuren | Tunesia | | | 2 | Short | | |
| **M-0284** | Belgium Royal Museum of Cantral Africs Tervuren | Tunesia | | | | | | |
| **M-0285** | Belgium Royal Museum of Cantral Africs Tervuren | Tunesia | | | 2 | Short | | |
| **M-0286** | Belgium Royal Museum of Cantral Africs Tervuren | Tunesia | | | | | | |
| **M-0287** | Belgium Royal Museum of Cantral Africs Tervuren | Tunesia | | | 2 | Short | | |

| | | | | | | | | |
|---|---|---|---|---|---|---|---|---|
| **11908** | Natural History Museum (Naturhistorisches Museum) – Vienna | Tunisia | | | | | |
| **9812** | Belgium Royal Museum of Natural History Brussels | Libya | | | 2 | Short | |
| **9813** | Belgium Royal Museum of Natural History Brussels | Libya | | | | | |
| **9814** | Belgium Royal Museum of Natural History Brussels | Libya | | | | | |
| **9815** | Belgium Royal Museum of Natural History Brussels | Libya | | | | | |
| **9816** | Belgium Royal Museum of Natural History Brussels | Libya | | | | | |
| **9817** | Belgium Royal Museum of Natural History Brussels | Libya | | | | | |
| **9818** | Belgium Royal Museum of Natural History Brussels | Libya | | | | | |
| **18044** | Belgium Royal Museum of Natural History Brussels | Libya | 30,766 | 17,783 | 1 | | |
| **18043** | Belgium Royal Museum of Natural History Brussels | Libya | 30,833 | 17,783 | 1 | | |
| **18042** | Belgium Royal Museum of Natural History Brussels | Libya | 30,833 | 17,783 | 2 | Short | |
| **18041** | Belgium Royal Museum of Natural History Brussels | Libya | 30,766 | 17,833 | 1 | Short | |
| **16995** | Belgium Royal Museum of Natural History Brussels | Libya | 21,963 | 24,820 | 2 | Short | |
| **16994** | Belgium Royal Museum of Natural History Brussels | Libya | 21,963 | 24,820 | 2 | Short | |
| **11908** | Natural History Museum (Naturhistorisches Museum) – Vienna | Tunisia | 34,466 | 8,716 | | | |
| **ZBSC 0169** | Morocco 2011 | Morocco | 32,894316 | -4,999255 | outgroup | Short | |
| **ZBSC 0170** | Morocco 2011 | Morocco | 32.894316 | -4.999255 | outgroup | Short/Long | ADRA2B, GHR, IRBP, VWF, DBX5, Agouti |
| **ZBSC 0171** | Morocco 2011 | Morocco | 32.895635 | -5.003515 | outgroup | Short/Long | |
| **ZBSC 0172** | Morocco 2011 | Morocco | 32.895635 | -5.003515 | outgroup | Short | |
| **ZBSC 0173** | Morocco 2011 | Morocco | 32.895635 | -5.003515 | outgroup | Short | |

| | | | | | | | |
|---|---|---|---|---|---|---|---|
| **ZBSC 0174** | Morocco 2011 | Morocco | 32.910272 | -5.032408 | outgroup | Short/Long | ADRA2B, GHR, IRBP, VWF, DBX5, Agouti |
| **ZBSC 0400** | Morocco 2011 | Morocco | 33.25945 | 2.69051 | outgroup | Short/Long | ADRA2B, GHR, IRBP, VWF, DBX5 |
| **10178** | Morocco 2012 | Morocco | 34,388593 | -3,009135 | outgroup | Short/Long | ADRA2B, GHR, IRBP, VWF, DBX5, MC1R, Agouti |
| **10233** | Morocco 2013 | Morocco | 32,939682 | -2,490725 | outgroup | Short/Long | ADRA2B, GHR, IRBP, VWF, DBX5, MC1R |
| **10234** | Morocco 2013 | Morocco | 32,820313 | -2,585565 | outgroup | Short/Long | ADRA2B, GHR, IRBP, VWF, DBX5, MC1R, Agouti |

**Table S5.** List of the primers and respective PCR conditions used in mitochondrial and nuclear DNA analysis.

| Locus | Size (bp) | Forward primer | | Reverse primer | | Ta (℃) | Reference |
|---|---|---|---|---|---|---|---|
| **Cytb** | 897 | Jac1Fw | GGACTCCCCATGACCTAT | Jac1Rv | TGCTGGTTTACAAGACCA | 55 | (4) |
| | | Jac4Fw | CAAACCCACTTAATACGC | Jac4Rv | CGAGAAGAGGGATACGAC | | |
| | 325 | Jac4Fw | CAAACCCACTTAATACGC | Jac1Rv | TGCTGGTTTACAAGACCA | | |
| **DBX5** | 317 | DBX5F | CAACAACTGTCCTCCACA | DBX5R | CATGATAATTTCTCCCATCTC | TD 60-50 (-0.5) | (5) |
| **ADRA2B** | 693 | J-adra2B1F | CTGGCGCTCGACGTGCTCTT | J-adra2B1R | AGCACCTGGCCACGGAGAGT | TD 68-64 (-0.5) | This study |
| **GHR** | 798 | J-ghr3F | ACAATGATGACTCTTGGGTTGAGT | J-ghr3R | AAGGGCAGGGCAGTTGCATT | | |
| **IRBP** | 1058 | J-irbp2F | GCGGCCATCCAGCAGGTAAT | J-irbp3R | CCGGCAGCACTGACACCTGA | | |
| **UWF** | 874 | J-UWF4F | ACGGATGCCTCGCTCAGCTC | J-UWF7R | CTCCAGTTCCTGCTGGTTGGCA | | (4) |
| **Agouti** | 545 | exon2F | GTCAGTACTCCGCCCTCTGG | exon2R | AGACAAGGAGCAAGCCATAGC | TD 60-65 (-0.5) | This study |
| **MC1R** | 1091 | F3 | GACACTAGAACCCTCCTGATG | R3 | GAATATCACCATCTCCCTCTGC | TD 55-60 (-0.5) | |

**Table S6**. List of the GenBank sequences used in *cytb* analysis.

| Identification | GB Accession number | Province, Country | Latitude | Longitude | Clade | Cytb Sequence | Reference |
|---|---|---|---|---|---|---|---|
| **D144** | JX885131 | Richard Toll, Senegal | 16,43 | -15,66 | 1 | Short/Long | (6) |
| **D145** | JX885132 | Thilé, Senegal | 16,51 | -15,08 | 1 | Short/Long | (6) |
| **D146** | JX885133 | Nbeika, Mauritania | 17,95 | -12,23 | 1 | Short/Long | (6) |
| **D147** | JX885134 | Nbeika, Mauritania | 17,95 | -12,23 | 1 | Short/Long | (6) |
| **D149** | JX885136 | Akjoujt, Mautitania | 19,74 | -14,37 | 1 | Short/Long | (6) |
| **D150** | JX885137 | Ayn El taya, Mauritania | 20,25 | -13,27 | 1 | Short/Long | (6) |
| **D151** | JX885138 | Ayn El taya, Mauritania | 20,25 | -13,27 | 2 | Short/Long | (6) |
| **D152** | JX885139 | El Mhaoudat, Mauritania | 22,97 | -12,00 | 2 | Short/Long | (6) |
| **D153** | JX885140 | El Mhaoudat, Mauritania | 22,97 | -12,00 | 2 | Short/Long | (6) |
| **D154** | JX885141 | Niafounke, Mali | 15,93 | -3,97 | 1 | Short/Long | (6) |
| **D155** | JX885142 | Niafounke , Mali | 15,93 | -3,97 | 1 | Short/Long | (6) |
| **D156** | JX885143 | Tsinsack, Mali | 16,73 | -2,96 | 1 | Short/Long | (6) |
| **D157** | JX885144 | Tsinsack, Mali | 16,73 | -2,96 | 1 | Short/Long | (6) |
| **D158** | JX885145 | Tidarmene, Mali | 17,02 | 2,11 | 1 | Short/Long | (6) |
| **D159** | JX885146 | Tidarmene, Mali | 17,02 | 2,11 | 1 | Short/Long | (6) |
| **D160** | JX885147 | Abeibara, Mali | 19,01 | 1,83 | 2 | Short/Long | (6) |
| **D161** | JX885148 | Abeibara, Mali | 19,01 | 1,83 | 2 | Short/Long | (6) |
| **D162** | JX885149 | Oued Chacheguerène, Mali | 19,70 | 0,00 | 1 | Short/Long | (6) |
| **D163** | JX885150 | Oued Chacheguerène, Mali | 19,70 | 0,00 | 2 | Short/Long | (6) |
| **D164** | JX885151 | Kreb in Karoua, Mali | 19,78 | 0,33 | 1 | Short/Long | (6) |
| **D165** | JX885152 | Babangata, Niger | 12,91 | 2,40 | 1 | Short/Long | (6) |
| **D166** | JX885153 | Gangara, Niger | 14,61 | 8,50 | 1 | Short/Long | (6) |
| **D168** | JX885155 | Gangara, Niger | 14,61 | 8,50 | 2 | Short/Long | (6) |

| | | | | | | | |
|---|---|---|---|---|---|---|---|
| **D169** | JX885156 | Gangara, Niger | 14,61 | 8,50 | 2 | Short/Long | (6) |
| **D170** | JX885157 | Gangara , Niger | 14,61 | 8,50 | 1 | Short/Long | (6) |
| **D171** | JX885158 | Goûgaram, Niger | 18,55 | 7,78 | 2 | Short/Long | (6) |
| **D172** | JX885159 | Al Baydah, Algeria | 33,69 | 1,01 | 1 | Short/Long | (6) |
| **D174** | JX885161 | Batna, Algeria | 35,32 | 5,83 | 2 | Short/Long | (6) |
| **D175** | JX885162 | Tebessa, Algeria | 35,40 | 8,12 | 2 | Short/Long | (6) |
| **D176** | JX885163 | Tebessa, Algeria | 35,40 | 8,12 | 2 | Short/Long | (6) |
| **D191** | JX885164 | Hamma, Tunisia | 33,95 | 9,63 | 2 | Short/Long | (6) |
| **D194** | JX885165 | Matmata, Tunisia | 33,55 | 9,96 | 2 | Short/Long | (6) |
| **D177** | JX885166 | Sbeitla, Tunisia | 35,22 | 9,12 | 1 | Short/Long | (6) |
| **D178** | JX885167 | Sbeitla, Tunisia | 35,22 | 9,12 | 1 | Short/Long | (6) |
| **D179** | GU433437 | Sbeitla, Tunisia | 35,22 | 9,12 | 2 | Short/Long | (7) |
| **D180** | GU433439 | Menzel Chaker, Tunisia | 34,96 | 10,36 | 2 | Short | (7) |
| **D182** | GU433422 | Nefta, Tunisia | 33,87 | 7,87 | 1 | Short | (7) |
| **D186** | GU433408 | Nefta, Tunisia | 33,87 | 7,87 | 1 | Short | (7) |
| **D187** | GU433409 | Douz, Tunisia | 33,45 | 9,02 | 1 | Short | (7) |
| **D188** | GU433410 | Douz, Tunisia | 33,45 | 9,02 | 1 | Short | (7) |
| **D189** | GU433424 | Douz, Tunisia | 33,45 | 9,02 | 1 | Short/Long | (7) |
| **D190** | GU433412 | Hamma, Tunisia | 33,95 | 9,63 | 1 | Short | (7) |
| **D192** | GU433413 | Hamma, Tunisia | 33,95 | 9,63 | 1 | Short | (7) |
| **D195** | GU433415 | Matmata, Tunisia | 33,55 | 9,96 | 1 | Short | (7) |
| **D197** | GU433427 | Matmata, Tunisia | 33,55 | 9,96 | 1 | Short/Long | (7) |
| **D198** | GU433411 | Matmata, Tunisia | 33,55 | 9,96 | 1 | Short | (7) |
| **D200** | GU433421 | Matmata, Tunisia | 33,55 | 9,96 | 1 | Short | (7) |
| **D201** | GU433441 | Matmata, Tunisia | 33,55 | 9,96 | 2 | Short/Long | (7) |
| **D202** | GU433418 | Matmata, Tunisia | 33,55 | 9,96 | 1 | Short/Long | (7) |
| **D203** | GU433435 | Matmata, Tunisia | 33,55 | 9,96 | 2 | Short/Long | (7) |
| **D205** | GU433438 | Matmata, Tunisia | 33,55 | 9,96 | 2 | Short | (7) |
| **D207** | GU433425 | Matmata, Tunisia | 33,55 | 9,96 | 1 | Short/Long | (7) |
| **D208** | GU433426 | Matmata, Tunisia | 33,55 | 9,96 | 1 | Short/Long | (7) |
| **D212** | GU433429 | Matmata, Tunisia | 33,55 | 9,96 | 1 | Short/Long | (7) |

| | | | | | | | |
|---|---|---|---|---|---|---|---|
| **D215** | GU433420 | Tataouine, Tunisia | 32,93 | 10,45 | 1 | Short/Long | (7) |
| **D209** | GU433423 | Matmata, Tunisia | 33,55 | 9,96 | 1 | Short/Long | (7) |
| **D213** | JX885168 | Medenine, Tunisia | 33,33 | 11,00 | 2 | Short | (6) |
| **D217** | JX885169 | Hun, Libya | 29,12 | 15,93 | 2 | Short/Long | (6) |
| **D218** | JX885170 | Hun, Libya | 29,12 | 15,93 | 2 | Short/Long | (6) |
| **D219** | JX885171 | Hun, Libya | 29,12 | 15,93 | 1 | Short/Long | (6) |
| **D220** | JX885172 | Hun, Libya | 29,12 | 15,93 | 1 | Short/Long | (6) |
| **D221** | JX885173 | Adiri, Libya | 27,53 | 13,19 | 1 | Short/Long | (6) |
| **D222** | JX885174 | Adiri, Libya | 27,53 | 13,19 | 1 | Short/Long | (6) |
| **D223** | JX885175 | Adiri, Libya | 27,53 | 13,19 | 1 | Short/Long | (6) |
| **D224** | JX885176 | Adiri, Libya | 27,53 | 13,19 | 1 | Short | (6) |
| **D225** | JX885177 | Birak, Libya | 27,54 | 14,24 | 1 | Short/Long | (6) |
| **D226** | JX885178 | Birak, Libya | 27,54 | 14,24 | 1 | Short/Long | (6) |
| **D227** | JX885179 | Birak, Libya | 27,54 | 14,24 | 1 | Short/Long | (6) |
| **D228** | JX885180 | Birak, Libya | 27,54 | 14,24 | 1 | Short/Long | (6) |
| **D229** | JX885181 | Jalu, Libya | 29,02 | 21,55 | 1 | Short/Long | (6) |
| **D230** | JX885182 | Jalu, Libya | 29,02 | 21,55 | 1 | Short/Long | (6) |
| **D231** | JX885183 | Tazirbu, Libya | 25,70 | 21,13 | 1 | Short/Long | (6) |
| **D232** | JX885184 | Tazirbu, Libya | 25,70 | 21,13 | 2 | Short/Long | (6) |
| **D233** | JX885185 | Burj El Arab, Egypt | 29,97 | 31,27 | 2 | Short/Long | (6) |
| **D234** | JX885186 | Burj El Arab, Egypt | 29,97 | 31,27 | 2 | Short/Long | (6) |
| **D235** | JX885187 | Burj El Arab, Egypt | 29,97 | 31,27 | 2 | Short/Long | (6) |
| **D236** | JX885188 | Abu Rauwash , Egypt | 29,66 | 31,23 | 2 | Short/Long | (6) |
| **D237** | JX885189 | Al Jizah, Egypt | 29,15 | 29,98 | 2 | Short/Long | (6) |
| **D239** | JX885191 | Al Minya, Egypt | 28,17 | 30,74 | 2 | Short/Long | (6) |
| **D240** | JX885192 | Al Minya, Egypt | 28,17 | 30,74 | 2 | Short/Long | (6) |
| **D241** | JX885193 | Sulaibiya, Kuwait | 29,27 | 47,71 | 2 | Short/Long | (6) |
| **D242** | JX885194 | Sulaibiya, Kuwait | 29,27 | 47,71 | 2 | Short/Long | (6) |
| **USNM483105** | KC663576 | Morocco | 28,77 | -10,23 | 2 | Short | (8) |
| **USNM482686** | KC663514 | Niger | 15,75 | 6,60 | 2 | Short | (8) |
| **USNM482681** | KC663512 | Niger | 17,37 | 6,72 | 1 | Short | (8) |

| | | | | | | | |
|---|---|---|---|---|---|---|---|
| **USNM482673** | KC663513 | Niger | 18,97 | 5,97 | 1 | Short | (8) |
| **USNM482671** | KC663526 | Niger | 16,55 | 6,87 | 2 | Short | (8) |
| **USNM482504** | KC663580 | Algeria | 23,17 | 5,12 | 1 | Short | (8) |
| **USNM482503** | KC663577 | Algeria | 22,93 | 5,42 | 2 | Short | (8) |
| **USNM482502** | KC663565 | Algeria | 22,63 | 5,73 | 2 | Short | (8) |
| **USNM482499** | KC663557 | Algeria | 23,57 | 5,12 | 1 | Short | (8) |
| **USNM482491** | KC663549 | Algeria | 26,87 | -0,97 | 1 | Short | (8) |
| **USNM482482** | KC663539 | Algeria | 30,05 | -2,22 | 1 | Short | (8) |
| **USNM482480** | KC663531 | Algeria | 32,46 | -0,58 | 1 | Short | (8) |
| **USNM475885** | KC663572 | Morocco | 32,68 | -3,08 | 2 | Short | (8) |
| **USNM475865** | KC663579 | Morocco | 30,30 | -5,93 | 2 | Short | (8) |
| **USNM475820** | KC663525 | Morocco | 32,15 | -1,25 | 1 | Short | (8) |
| **USNM475797** | KC663524 | Morocco | 31,83 | -4,58 | 1 | Short | (8) |
| **USNM475783** | KC663574 | Morocco | 32,50 | -2,05 | 2 | Short | (8) |
| **USNM475780** | KC663575 | Morocco | 31,95 | -3,55 | 1 | Short | (8) |
| **USNM475764** | KC663573 | Morocco | 32,12 | -2,85 | 2 | Short | (8) |
| **USNM475761** | KC663578 | Morocco | 31,90 | -4,48 | 2 | Short | (8) |
| **USNM401212** | KC663571 | Mauritania | 21,52 | -13,05 | 1 | Short | (8) |
| **USNM350066** | KC663536 | Egypt | 30,08 | 31,58 | 2 | Short | (8) |
| **USNM342084** | KC663515 | Sudan | 15,23 | 36,39 | 1 | Short | (8) |
| **USNM342040** | KC663521 | Egypt | 29,70 | 32,35 | 1 | Short | (8) |
| **USNM342034** | KC663529 | Egypt | 28,54 | 30,57 | 1 | Short | (8) |
| **USNM342033** | KC663540 | Egypt | 28,32 | 31,12 | 2 | Short | (8) |
| **USNM342030** | KC663528 | Egypt | 27,14 | 31,38 | 2 | Short | (8) |
| **USNM342028** | KC663543 | Egypt | 27,22 | 30,80 | 1 | Short | (8) |
| **USNM325828** | KC663550 | Libya | 29,59 | 24,86 | 1 | Short | (8) |
| **USNM325821** | KC663569 | Libya | 31,19 | 16,40 | 1 | Short | (8) |
| **USNM325819** | KC663566 | Libya | 30,55 | 18,47 | 2 | Short | (8) |
| **USNM325805** | KC663563 | Libya | 29,57 | 24,70 | 1 | Short | (8) |
| **USNM325802** | KC663562 | Libya | 29,75 | 24,55 | 1 | Short | (8) |
| **USNM325789** | KC663570 | Libya | 25,75 | 21,15 | 1 | Short | (8) |

| | | | | | | | |
|---|---|---|---|---|---|---|---|
| **USNM325774** | KC663561 | Libya | 29,25 | 21,23 | 1 | Short | (8) |
| **USNM325770** | KC663560 | Libya | 32,42 | 13,05 | 1 | Short | (8) |
| **USNM322811** | KC663554 | Libya | 27,53 | 13,20 | 1 | Short | (8) |
| **USNM322809** | KC663553 | Libya | 27,55 | 14,25 | 1 | Short | (8) |
| **USNM322803** | KC663568 | Libya | 27,00 | 14,45 | 1 | Short | (8) |
| **USNM322798** | KC663558 | Libya | 25,90 | 13,89 | 1 | Short | (8) |
| **USNM322788** | KC663556 | Libya | 24,95 | 10,21 | 1 | Short | (8) |
| **USNM322770** | KC663555 | Libya | 26,77 | 14,00 | 1 | Short | (8) |
| **USNM322767** | KC663559 | Libya | 27,22 | 14,66 | 1 | Short | (8) |
| **USNM322762** | KC663564 | Libya | 29,09 | 15,90 | 1 | Short | (8) |
| **USNM321864** | KC663551 | Libya | 30,75 | 11,52 | 2 | Short | (8) |
| **USNM321863** | KC663567 | Libya | 32,06 | 11,35 | 1 | Short | (8) |
| **USNM319773** | KC663552 | Libya | 24,18 | 23,32 | 1 | Short | (8) |
| **USNM317068** | KC663546 | Egypt | 28,56 | 33,96 | 2 | Short | (8) |
| **USNM317065** | KC663527 | Egypt | 24,01 | 32,83 | 1 | Short | (8) |
| **USNM317059** | KC663522 | Egypt | 30,41 | 30,60 | 2 | Short | (8) |
| **USNM317050** | KC663534 | Egypt | 30,10 | 31,58 | 1 | Short | (8) |
| **USNM317049** | KC663519 | Egypt | 30,63 | 29,84 | 1 | Short | (8) |
| **USNM317047** | KC663518 | Egypt | 29,49 | 30,40 | 1 | Short | (8) |
| **USNM317041** | KC663520 | Egypt | 30,22 | 30,90 | 1 | Short | (8) |
| **USNM317028** | KC663541 | Egypt | 25,26 | 32,46 | 1 | Short | (8) |
| **USNM317020** | KC663537 | Egypt | 30,50 | 30,79 | 2 | Short | (8) |
| **USNM317018** | KC663533 | Egypt | 30,41 | 30,60 | 1 | Short | (8) |
| **USNM317017** | KC663542 | Egypt | 25,67 | 32,77 | 1 | Short | (8) |
| **USNM317015** | KC663544 | Egypt | 31,52 | 25,61 | 2 | Short | (8) |
| **USNM317014** | KC663532 | Egypt | 22,30 | 36,54 | 1 | Short | (8) |
| **USNM317013** | KC663545 | Egypt | 22,27 | 36,40 | 1 | Short | (8) |
| **USNM317012** | KC663530 | Egypt | 22,53 | 36,23 | 1 | Short | (8) |
| **USNM297613** | KC663516 | Sudan | 19,87 | 37,18 | 1 | Short | (8) |
| **USNM297612** | KC663517 | Sudan | 19,54 | 37,19 | 1 | Short | (8) |
| **USNM283260** | KC663538 | Egypt | 30,31 | 32,28 | 2 | Short | (8) |

| | | | | | | | |
|---|---|---|---|---|---|---|---|
| **USNM282539** | KC663535 | Egypt | 30,09 | 31,43 | 2 | Short | (8) |
| **2002512** | JX885154 | Babangata, Niger | 12,91 | 2,40 | 2 | Short/Long | (6) |
| **2002274** | JX885160 | Goûgaram, Niger | 18,55 | 7,78 | 1 | Short/Long | (6) |

**Table S7.** Evolutionary models of each locus used for phylogenetic and demographic analyses. Calculations made with jModelTest.

| | **Phylogenetics analyses/ Species tree inference** | **EBSP analyses** | |
|---|---|---|---|
| | | ***J. jaculus*** | ***J. hirtipes*** |
| **Cytb** | HKY+I+G | HKY+I+G | HKY+I |
| **DBX5** | HKY | - | - |
| **DBX5 without recombination** | HKY | HKY | HKY |
| **ADRA2B** | HKY+I | HKY+I | HKY+I |
| **GHR** | HKY | HKY | HKY |
| **IRBP** | HKY+I | - | - |
| **UWF** | HKY+I | - | - |
| **UWF without recombination** | HKY+I | HKY+I | HKY+I |
| **MC1R** | HKY+I | GTR | GTR |
| **Agouti** | HKY+I | HKY | HKY |

**Table S8.** Information on the microsatellites used in each multiplex and individual reaction and respective PCR conditions.

| Multiplex | Locus | Size(bp) | Forward Primer | Reverse primer | Repeat number | Motif | Volume (in 100µL) | Tail | Ta (°C) | Nº of cycles |
|---|---|---|---|---|---|---|---|---|---|---|
| 1 | **Jac01** | 101 | GATGGCTGTAGCTGTCTGGG | GAACCATAGTAAGATAACAGCATGG | 14 | tg | 0.8 | FAM | TD 57-51 (-0.5) | 40 |
| | **Jac02** | 144 | CACAGACTGAAACCGTGAGC | CCAAAGAGGAGGCACAGAAG | 12 | ac | 0.8 | FAM | | |
| | **Jac04** | 105 | ATCAGCCTCTCAGCCTTCTG | ACTGCAGGCTCTCGTGTTCT | 11 | ga | 0.8 | VIC | | |
| | **Jac23** | 234 | AACAAGAATGAATACATGGGGA | TAGGTGTGCACCACCACACT | 11 | ac | 1.04 | VIC | | |
| | **Jac07** | 95 | TTCATGCCAAGTTCAAAGGC | ATCGCAACAAGAAAGATGGC | 18 | ac | 0.8 | NED | | |
| | **Jac08** | 140 | CAAGGAACGTGCCTGACTTT | TAGCGTCCCTGTTTTCCTTC | 12 | ac | 2.0 | NED | | |
| | **Jac24** | 141 | ACAGTCCCCTTTAACATGATAGTC | CTTCTGTTAGTAGCTGAGACATGATT | 16 | gt | 2.0 | PET | | |
| 2 | **Jac11** | 111 | CCACCTTCTATCATAAATACACAGTGA | GGCCGTTGTATGTGAGTCAA | 21 | ca | 2.0 | FAM | TD 55-49 (-0.5) | 45 |
| | **Jac27** | 140 | GGTGTAACCCTGACCTAATCC | TGTCTATGTAACTCATGACCAAGAA | 14 | ac | 2.0 | VIC | | |
| | **Jac16** | 190 | TCTGTCTTAGGAATATTGGGCA | TGTCTTGATTTCTTCTCTGTTTTATTG | 12 | ac | 0.8 | VIC | | |
| | **Jac18** | 176 | GCCCCAATATTTCATGTTTCA | GGCTTCTGGAGTTCATTTGC | 20 | ca | 1.0 | NED | | |
| Individual reactions | **Jac12** | 166 | ACCTGCCAGCAACGATGT | GGCCGTTGTATGTGAGTCAA | 20 | tg | - | FAM | TD 58-51 (-0.5) | 40 |
| | **Jac37** | 190 | TGTCACATGAAATTAATAGGGCAT | TCTTTGGTATTCCTCAACTCGG | 11 | atgaa | - | PET | | |

## Figures

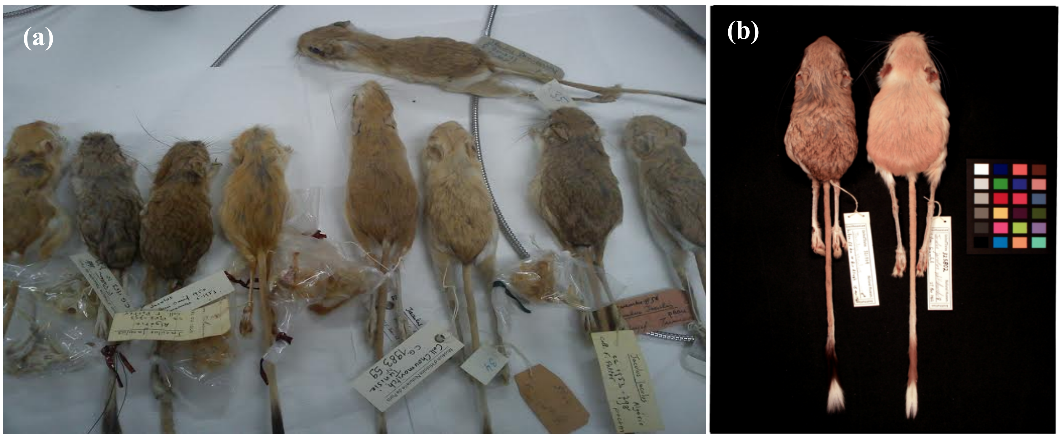


**Figure S1.** Phenotypic variation between the two lineages described. (a) A photography demonstrating the wide range of fur colour of the two species; (b) A photography representing the colour variation observed within African jerboas, defining two putative cryptic species (8).

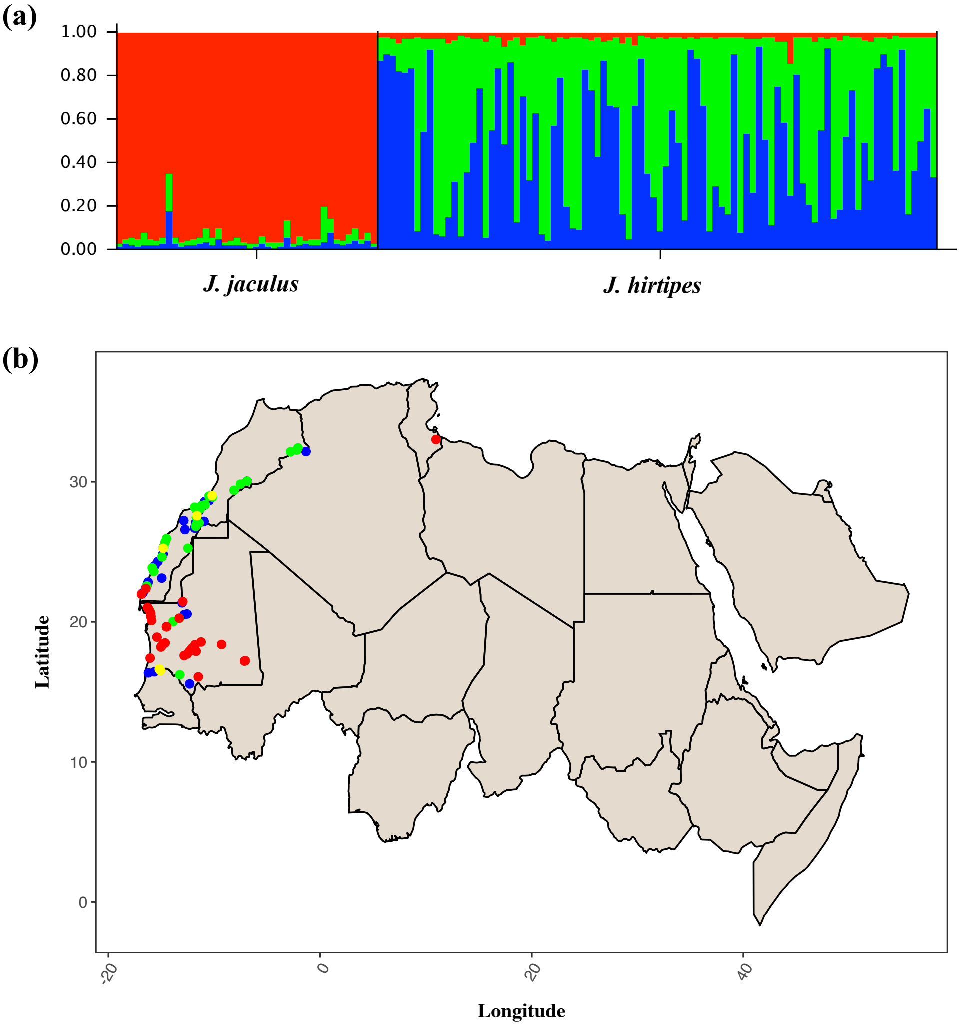


**Figure S2. (a)** Structure bar plot of Bayesian assignments of the individuals to the respective cluster for K=3. Vertical bars indicate individuals and the colours within each bar denote for the probability of membership of each specimen to a cluster. **(b)** Geographic distribution of the specimens used in (a) plotted using the R packages "ggplot2" (9) and

“ggmaps” (10). Red, blue and green dots correspond to the individuals with a proportion of membership to the respective cluster in (a) higher than 50%, and yellow dots correspond to the specimens with mixed memberships in (a), where the proportion of membership is lower than 50% in each cluster.

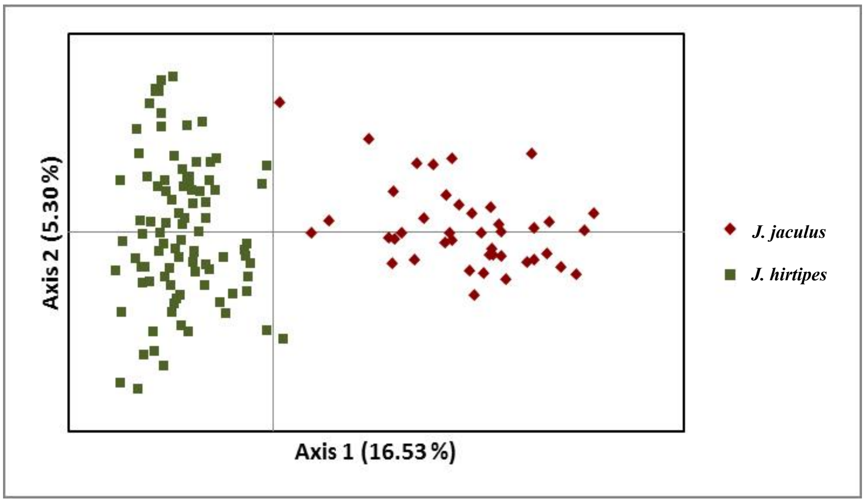


**Figure S3.** Principal coordinate analysis (PCA) based on the individual-by-individual genetic distances.

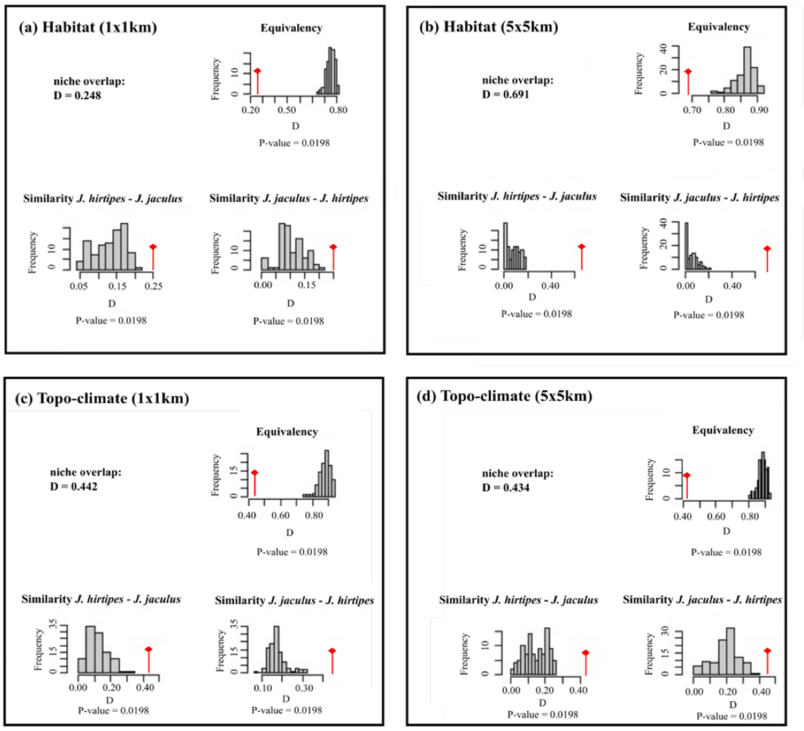


**Figure S4.** Representation of Schoener’s D Index value (D) for niche overlap (red sign), similarity and identity tests for habitat (at 1x1km and 5x5km scales for (a) and (b) respectively) and topo-climate variables (at 1x1km and 5x5km scales for (c) and (d) respectively). P-values are presented on the bottom of each analysis.

(a)

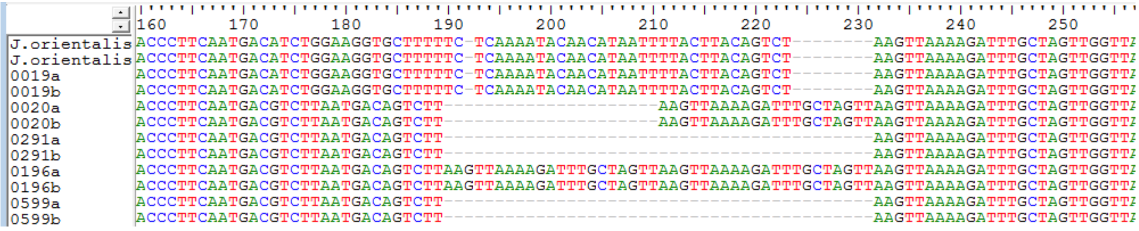

(b)

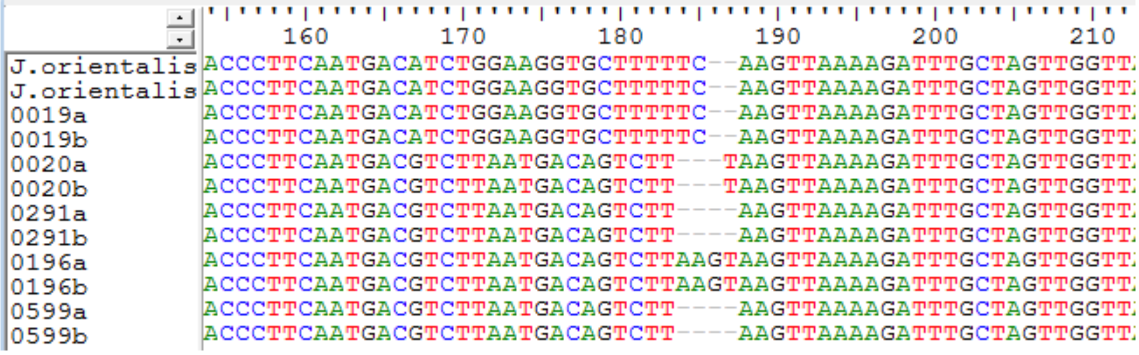

**Figure S5.** Sequence alignment of the DBX intron where the insertion/deletion polymorphisms can be observed (a). Indels were coded so that a more robust approximation of the mutational steps could be made for the phylogenetic network's reconstruction. In (b) is displayed the alignment after coding the polymorphisms.

## References


1. Taberlet P, Luikart G, Waits LP. Noninvasive genetic sampling: Look before you leap. Trends Ecol Evol. 1999;14(8):323–7.

2. Vallone PM, Butler JM. AutoDimer: A screening tool for primer-dimer and hairpin structures. Biotechniques. 2004;37(2):226–31.

3. Evanno G, Regnaut S, Goudet J. Detecting the number of clusters of individuals using the software STRUCTURE: A simulation study. Mol Ecol. 2005;14(8):2611–20.

4. Boratyński Z, Brito JC, Mappes T. The origin of two cryptic species of African desert jerboas (Dipodidae: Jaculus). Biol J Linn Soc. 2012;105(2):435–45.

5. Hellborg L, Ellegren H. Low Levels of Nucleotide Diversity in Mammalian Y Chromosomes. Mol Biol Evol. 2004;21(1):158–63.

6. Ben Faleh A, Granjon L, Tatard C, Boratyński Z, Cosson JF, Said K. Phylogeography of two cryptic species of African desert jerboas (Dipodidae: Jaculus). Biol J Linn Soc.. 2012;107(1):27–38.

7. Ben Faleh A, Cosson JF, Tatard C, Othmen A Ben, Said K, Granjon L. Are there two cryptic species of the lesser Jerboa jaculus jaculus (Rodentia: Dipodidae) in tunisia? evidence from molecular, morphometric, and cytogenetic data. Biol J Linn Soc. 2010;99(4):673–86.

8. Boratyński Z, Brito JC, Campos JC, Karala M, Mappes T. Large spatial scale of the phenotype-environment color matching in two cryptic species of African desert jerboas (Dipodidae: Jaculus). PLoS One. 2014;9(4):e94342.

9. Wickham H. ggplot2 - Elegant Graphics for Data Analysis. Springer. 2016.

10. Kahle D, Wickham H. ggmap: Spatial Visualization with ggplot2. R J. 2013;5(1):144–61.
